# Interventions to reduce sedentary behaviour in community-dwelling older adults: a mixed-method review

**DOI:** 10.1186/s12966-025-01835-3

**Published:** 2025-11-12

**Authors:** Ragy Tadrous, Anne Forster, Amanda Farrin, Peter Coventry, Andrew Clegg

**Affiliations:** 1 Academic Unit for Ageing and Stroke Research, University of Leeds, Leeds, England; 2 Bradford Institute for Health Research, University of Leeds, Bradford, UK; 3 Clinical Trials Research Unit, University of Leeds, Leeds, England; 4 Department of Health Sciences, University of York, York, England; 5 Better Health & Care Hub, King’s College London, London, SE5 9RS England

**Keywords:** Sedentary behaviour, Mixed-method review, Older adults, Behavioural change techniques, Thematic synthesis, Meta-analysis

## Abstract

**Background:**

Older adults are the fastest-growing and most sedentary group in society. As sedentary behaviour is associated with deleterious health outcomes, reducing sedentary time may improve overall well-being. This mixed-methods systematic review aimed to systematically review quantitative and qualitative studies examining interventions to reduce sedentary behaviour in community-dwelling older adults (aged ≥ 65 years).

**Methods:**

Medline, Embase, Cochrane Central Register of Controlled Trials, Web of Science, Cinahl, SportDiscus, and PEDRO were searched from inception to July 2025. We included quantitative studies (randomised-controlled trials (RCTs) and cluster RCTs), qualitative studies (semi-structured interviews or focus groups), and mixed-method studies exploring interventions to reduce sedentary behaviour in community-dwelling older adults. Studies were appraised using the Mixed Method Appraisal Tool. Quantitative evidence was meta-analysed; qualitative evidence was thematically synthesised, with both combined in a mixed-method synthesis. The Behaviour Change Techniques employed were charted and analysed.

**Results:**

Fifty-six studies (16 RCTs, 30 qualitative, and 10 mixed-method studies) were included. When pooled, interventions reduced sedentary behaviour by 27.53 min/day (95% CI: − 57.43 to 2.37), with greater reductions observed via self-report (–83.65 min/day) than device measures (–11.61 min/day). Using ≥ 11 BCTs (-24.01 min/day) was more effective than using 1–10 **(**9.24 min/day). Analytical themes included what sitting means to older adults, expectations of ageing, and social influence in older adults. The mixed-method synthesis identified that existing interventions are limited by recruited samples that are not representative of the wider population of older adults, and intervention design and outcome measurement selection that is not consistent with older adults’ priorities.

**Conclusions:**

Interventions to reduce sedentary behaviour in community-dwelling older adults are somewhat effective at reducing sedentary time. Future research should focus on inclusive recruitment strategies to recruit underrepresented populations, incorporate outcome measures valued by older adults, and align intervention content with their preferences.

**PROSPERO registration number:**

CRD42021264954.

**Supplementary Information:**

The online version contains supplementary material available at 10.1186/s12966-025-01835-3.

## Introduction

Sedentary behaviour (SB) is defined as “any waking behaviour characterized by an energy expenditure ≤ 1.5 METs while in a sitting or reclining posture” [1]. SB is associated with adverse health outcomes, including poor self-reported health, decreased physical function, and higher healthcare usage [2, 3]. Older adults are the fastest-growing demographic, and with approximately 67% of older adults spending >8.5 h per day sedentary, they are also the most sedentary [4, 5]. As such, the World Health Organisation highlighted the importance of limiting SB in adults (including older adults), replacing sedentary time with physical activity (PA), and performing moderate-vigorous intensity physical activity (MVPA) to reduce the detrimental effects of prolonged sedentary behaviour on health and wellbeing [6].

Several quantitative reviews have explored interventions to reduce SB in older adults. A 2018 review was the first to explore interventions to reduce SB in non-working older adults, and their findings suggest that behavioural interventions can potentially reduce sitting time [7]. With only six studies included, meta-analyses could not be completed due to a small sample size. A review by Shrestha et al. [8] explored the effectiveness of interventions for reducing non-occupational sedentary behaviour in adults and older adults, but did not find any randomised controlled trials (RCTs) where the mean age of participants was above 60 years. A scoping review by Petrusevski et al. [9] narratively explored interventions to reduce SB in this population, highlighting the importance of multi-component interventions (including components such as education and activity monitoring) and the underrepresentation of adults aged ≥ 75 years.

A later review by Chase et al. [10] reported that interventions to reduce SB can reduce SB, but the overall effect size was small. This review included PA interventions and did not conduct subgroup analyses, and consequently, the effects of interventions that aimed to reduce SB cannot be discerned from interventions that aimed to reduce SB and increase PA. Similarly, a 2021 Cochrane review explored this topic [11], but four of the seven interventions included aimed to increase PA and reduce SB [12–15]. Without subgroup analysis, the effectiveness of interventions to reduce SB in older adults remains unclear. Ahmed et al. [16] systematically reviewed the behaviour change techniques (BCTs) in interventions that aimed to increase PA or reduce SB in community-dwelling older adults aged 50–70. This review focused primarily on interventions which aimed to increase PA and did not target the oldest old.

Similarly, several qualitative reviews focus on SB in older adults. Previous reviews have explored older adults’ experiences with PA interventions [17], and adults’ experiences of interventions to reduce SB [18], with four of the 30 included studies conducted with older adults. A thematic synthesis by Compernolle et al. [19] explored the perception of older adults towards SB and described the habitual nature of SB and the importance of enjoyment and convenience when attempting to reduce SB in this population. Ramalho et al. [20] provided an updated review of the qualitative literature in 2023, and explored perspectives of SB, daily routines, advantages and disadvantages surrounding SB, barriers and facilitators to reducing SB, and perceptions of interventions to reduce SB. However, their review did not focus solely on community-dwelling older adults, including studies conducted with people in assisted living facilities [21–24]. Older adults in assisted living facilities may be more sedentary due to limited need for engagement in light-intensity physical activities (LIPA) such as cleaning or preparing meals [25]. As such, they may be more sedentary and less active than older adults living in the community [26–28]. Additionally, their review did not include mixed-method studies, which can provide valuable insights into older adults’ perceptions of interventions to reduce SB. As such, the perceptions of community-dwelling older adults aged >65 years towards SB and interventions to reduce SB have yet to be fully elucidated.

These reviews provide an understanding of SB and interventions to reduce SB in community-dwelling older adults. However, given the rapidly expanding nature of the literature, updates on the current state of the research can help guide evidence-based practice. Mixed-method reviews combine studies from different research traditions that focus on the same topic [29], integrating the quantitative estimate of benefit and harm with the qualitative understanding of people’s lives [30]. Furthermore, mixed-method reviews provide a novel interpretation of the data that would not have been achieved had the reviews been completed separately [31]. This mixed-method review aimed to:

i. Synthesise the effectiveness of interventions to reduce SB in community-dwelling older adults aged ≥ 65 years.
ii. Chart and analyse the effectiveness of BCTs of included interventions.
iii. Explore the effect of interventions to reduce SB on secondary outcome measures including health-related quality of life (HRQoL), MVPA, LIPA, physical performance, body composition and cardiometabolic biomarkers.
iv. Explore sedentary activities and barriers and facilitators to reducing SB in this population.
v. Explore older adults’ attitudes towards SB and interventions to reduce SB.
vi. Integrate quantitative and qualitative findings to explore the suitability of existing interventions to reduce SB in community-dwelling older adults.

## Methods

A protocol detailing the search strategy and review methodology was registered on Prospero (www.crd.york.ac.uk/prospero/) in June 2021 (Identification number: CRD42021264954). The review followed the Preferred Reporting Items of Systematic Reviews and Meta-analysis (PRISMA) guidelines [32].

### Eligibility criteria

#### Research type

Quantitative, qualitative, and mixed-method studies were considered. Randomised controlled trials (RCTs) and cluster RCTs were eligible. Qualitative studies using qualitative research methodologies, including semi-structured interviews or focus groups were eligible. Qualitative data gathered and analysed through quantitative methodologies were excluded (e.g., surveys analysed quantitatively). Mixed-method studies were considered if the qualitative and quantitative components could be separately extracted and warranted inclusion in their respective syntheses.

### Phenomenon of interest

Studies that explored interventions to reduce SB, or older adults’ attitudes towards SB and interventions to reduce SB were included. SB was defined as any waking behaviour characterised by an energy expenditure of ≤ 1.5 METS whilst sitting or lying down. Studies that aimed to increase PA and reduce SB were also included but analysed and reported separately.

### Population

This review included studies that recruited community-dwelling older adults aged ≥ 65 years. Community-dwelling was defined as older people who live at home, with studies in residential/nursing homes excluded. Studies conducted in older adults with multiple comorbidities or specific clinical populations (e.g., older adults with obesity) were eligible.

### Outcome of interest (quantitative studies)

Sedentary time, measured using devices such as accelerometers or inclinometers or self-reported using validated questionnaires such as the Measure of Older Adults’ Sedentary Time [33].

### Comparator

Controls without interventions or interventions which did not target SB were eligible.

### Electronic searches

A search strategy (supplemental) was developed in collaboration with an Information Specialist (DA). Search strings were developed for the following databases: Medline, Embase, Cochrane Central Register of Controlled Trials, Web of Science, Cinahl, SportDiscus and PEDRO.

### Data management and selection

Following the search, references were deduplicated and uploaded to the online systematic review tool, Covidence (www.covidence.org). One reviewer screened all titles and abstracts (RT), with a second reviewer screening approximately 20% of titles (*n* = 10,100), during which there 141 conflicts, a proportional agreement of 98.60%. The full texts of potentially eligible studies to determine eligibility for inclusion were screened by two reviewers (RT and CQ/PA), with disagreements were resolved through discussion until an agreement was reached.

### Data extraction and appraisal

Data were extracted using a modified version of the Cochrane data extraction tool [34] by one assessor (RT). Extracted data included the publication year, country, research design, age, sample size, gender, and populations recruited. The data collection and analysis method, and descriptions of the intervention and control were extracted for qualitative and quantitative articles, respectively. Methodological quality of included articles was assessed using the Mixed-Method Appraisal Tool (MMAT) by one reviewer (RT) and discussed with the other reviewers [35].

### Quantitative synthesis

Included studies were synthesised narratively. Total sedentary time was identified by pooling device-measured and self-reported sedentary time; however, subgroup analyses were conducted to enable separate reporting. Time spent in specific sedentary activities (e.g., television viewing), sit-to-stand transitions, sedentary breaks, and time spent in different sedentary bout lengths were also pooled where possible. The following outcomes were also meta-analysed where possible: MVPA, LIPA, physical performance, HRQoL, body composition, blood pressure, and cardiometabolic blood markers. Analyses were stratified according to the type of intervention (SB versus PA and SB) and measurement of SB (self-rated versus device-measured). Pooled effects were based on mean between-groups difference for the end-of-intervention final endpoint and a random-effects meta-analyses were conducted using the software Review Manager (RevMan) [36]. Where possible, missing values (e.g., standard deviations (SDs)) were calculated from available data (confidence intervals or standard errors) [34]. Study authors were contacted to obtain missing data. Effect sizes were assessed using 95% confidence intervals, with attention to the direction and magnitude of effects to determine whether they were positive or negative. Statistical significance was set at *p* < 0.05. Heterogeneity was determined using I^2^ values, with values approaching 25%, 50%, and 75%, representing low, moderate, and high proportions of variability due to between-study heterogeneity, respectively [37]. Publication bias was assessed using Egger’s test, Duval and Tweedie’s trim and fill analysis and funnel plots using Comprehensive Meta-Analysis [38].

### Qualitative synthesis

A thematic synthesis was conducted by one reviewer (RT) with the research team engaging in regular discussions throughout the analytical process, which helped shape the interpretation of data and provided alternative perspectives. The thematic synthesis of participant quotes was guided by the three-stage process outlined by Thomas and Harden [39]: (i) line-by-line coding of text; (ii) development of descriptive themes; (iii) generation of analytical themes. The text was coded, and themes were developed using QSR NVivo 14. A deductive approach was adopted to categorise the activities performed in sitting to the ecological model of SB according to leisure-related, occupation-related, household-related or transport-related SBs [34]. A similar approach was adopted to: (i) extract and categorise the barriers and facilitators present to reducing SB according to the capability, opportunity, and motivation model of behaviour (COM-B) [40] and (ii) extract and match the intervention components described with the reported BCT [41]. Where BCTs were not reported, the intervention descriptions were extracted and charted to the most-appropriate BCT by one reviewer (RT) and checked by another reviewer (JW). The lead author had undertaken certified training in the BCT taxonomy (available at www.bct-taxonomy.com).

### Mixed-method synthesis

Following a parallel-results convergent design, quantitative and qualitative findings were reported separately and integrated narratively in the discussion [29]. Quantitative and qualitative findings were juxtaposed through a matrix table.

## Results

### Screening

**Fig. 1:**
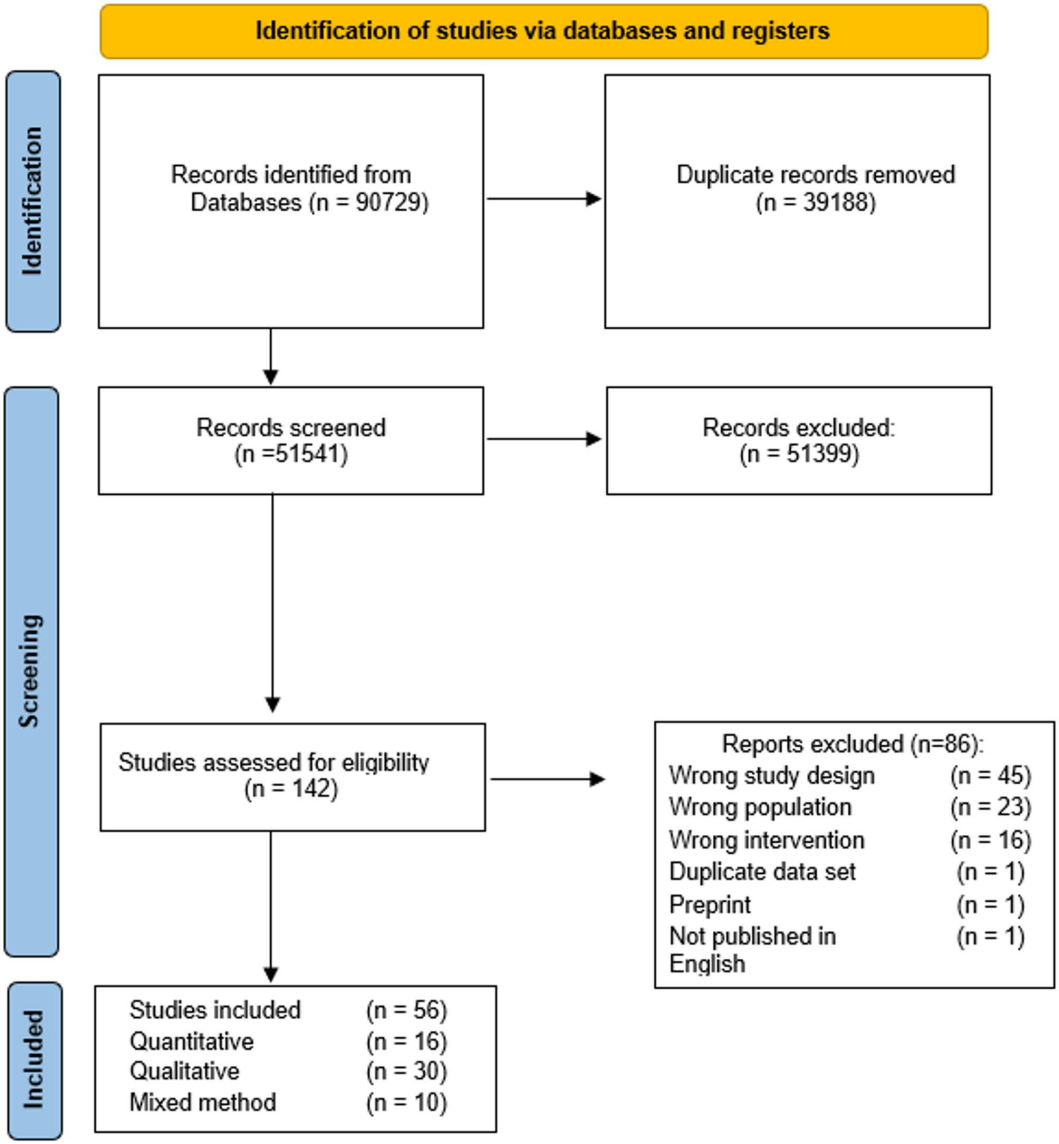
Flow diagram of studies screened for eligibility

Qualitative and quantitative searches were run in January 2021, rerun in July 2025, and identified 90,729 articles. When retrieved articles were deduplicated, 51,541 unique articles were identified and assessed for eligibility. Full texts of 142 articles were screened to determine eligibility, with 86 ineligible and 56 articles included (Fig. 1).

### Study characteristics

The characteristics of the included studies are outlined in Table 1. A total of 56 articles were included. The studies represent a total sample of 2793 older adults, of which 63.69% were female (*n* = 1779) with a weighted mean age of 72.15 years (SD 8.57). This review included 16 RCTs [13, 15, 42–55], 30 qualitative studies [55–84], and ten mixed-method studies [14, 85–93].

Twenty-one studies [13–15, 42–55, 85, 86, 91, 92] were included in the quantitative synthesis and represented 1937 older adults (66.49% female; *n* = 1288). The weighted mean age of participants was 71.62 years (SD 8.57 years). Included studies originated from eight countries, with ten conducted in America [15, 42, 43, 45, 47, 49–52, 91], four in the United Kingdom [14, 85, 86, 92], and one study conducted in each remaining country (Table 1). Fourteen studies were classified as interventions to reduce SB [42–46, 49–53, 85, 86, 91, 93], and seven studies were classified as interventions to increase PA and reduce SB [13–15, 47, 48, 54, 55].

Forty studies [14, 55–93] were included in the qualitative review and represented 1078 older adults (56.78% female; *n* = 619). The weighted mean age of participants was 72.48 years (SD 7.91 years). Included articles originated from six countries, with 20 studies conducted in the United Kingdom [14, 57, 58, 62, 65, 66, 69–72, 76, 77, 79, 80, 85, 86, 88–90, 93], and six in Canada [60, 68, 78, 81, 82, 84]. Remaining studies were conducted in America [63, 67, 75, 91], Sweden [61, 64, 73, 74], Australia [59, 83], Belgium [87], Sweden [64], or Singapore [55], and one multi-centre study conducted in several European countries [56].

**Table 1:** Study characteristics of included articles

Quantitative Articles	Year	Country	Design	Mean Age (years (SD))	Sample Size	Gender (F)		Population	Intervention	Intervention Duration (Weeks)	Follow-Up (Weeks)	Control
Aunger et al.	2020	United Kingdom	Mixed-Method: Feasibility RCT	73.14 (5.78)	35	20	57.10%	Older adults >60 years oldListed for elective hip or knee surgery	SB: Intervention based on Self-Determination Theory, involved techniques to reduce SB, including motivational interviewing, setting of behavioural goals.	6	6 weeks post-surgery	Regular orthopaedic care
Bailey et al.	2024	United Kingdom	Mixed-Method: Feasibility RCT	74 (6)	60	40	67%	Older adults with very mild or mild frailty	SB: Remote intervention with a psychoeducation workbook, tailored feedback, wearable device, health coaching and peer support	26 (6 months)	Intervention completion	Standard healthcare
Barone Gibbs et al.	2016	America	RCT	67.9 (6.54)	38	27	71.10%	Older adults ≥ 60 years old	SB: Behavioural change intervention targeting reducing SB	12	Intervention completion	150 min of MVPA/week
Blair et al.	2021	America	RCT	69.6 (4.8)	54	30	55.60%	Older adultsCancer survivors	SB: Home-based mobile health (mHealth) intervention to interrupt and replace sedentary time with standing and stepping	13	Intervention completion	Waitlist Control
Cheng et al.	2022	Australia	RCT	73.48 (9.48)	65	33	50.80%	Adults with COPD awaiting Pulmonary Rehab	SB: Behavioural change intervention delivered by physiotherapists through education, guided goals setting and real-time feedback on SB	6	Intervention completion	Phone calls to monitor health status
Crombie et al.	2021	America	RCT	74.02 (7.21)	56	49	87.50%	Healthy community-dwelling older adults	SB: Stand Up and Move More: based on self-regulation and social cognitive theories. Behavioural intervention consisting of group sessions – information provision, problem solving, goal setting, and action planning.	4	8 weeks following completion	Waitlist Control
English et al.	2016	Australia	RCT	66.9 (12.7)	33	13	39.40%	Stroke survivors	SB: Four Counselling Sessions: sit less, move more’	7	1 week following cessation	Calcium supplement & attention matching
Fanning et al.	2016	America	RCT	70.67 (4.72)	221	171	77.40%	Inactive Older adults (>65 years).	PA + SB: Exercise DVDs, provided information on replacing sitting behaviours.	26 (6 months)	6 months	“Healthy Aging DVD”
Kallings et al.	2009	Sweden	RCT	67.5 (NR)	101	58	57.40%	BMI between 25 and 40 kg/m2) and abdominal obesity	PA + SB: Counselling, written prescription for activity. Encouraged to reduce time spent in SB.	26 (6 months)	Intervention completion	Low-intensity intervention, general PA information
Leitzelar et al.	2024	America	Mixed-Method: Feasibility RCT	70.75 (9.07)	18	NR	NR	African American Older Adults	SB: Stand Up and Move More: based on self-regulation and social cognitive theories. Behavioural intervention consisting of group sessions – information provision, problem solving, goal setting, and action planning.	4	Intervention Completion	Stress management workshop
Lyden et al.	2021	America	RCT	69 (13)	106	45	42.50%	CKD Stage 2–4, BMI 25.0–39.9 kg/m2, gait speed 0.7 m/s, walk 250 m in the 6-minute walk test	SB: Sit Less, Interact, Move More intervention: Education and personalised feedback	24	Intervention completion	150 min of MVPA/week
Maher et al.	2017	America	Cluster RCT	76.9 (9.2)	41	38	92.7%	Older adults	SB: Behavioural change intervention: Group sessions, define SB, create awareness, enhance self-efficacy, action planning, behavioural goal setting	2	Intervention completion	Education about reducing social isolation
Olanrewaju et al.	2022	United Kingdom	Mixed-Method: Feasibility RCT	74.6 (7.5)	23	9	39.1%	Community-dwelling adults aged 50+ years with mild cognitive impairment	SB: Walking, addressing unpleasant sensations, education, cueing, remote delivery of intervention, educational booklet, online coaching, health coaching	12	Intervention completion	Written information on the benefits of increasing activity levels
Owari et al.	2019	Japan	RCT	71.83 (5.52)	86	56	65.10%	Community-dwelling older adults	PA + SB: ‘Active Guide’ brochure published by the Ministry of Health, Labour, and Welfare (2013) and additional documents related to the benefits of reducing SB.	52	Intervention completion	Received only the results of their baseline examination.
Roberts et al.	2019	America	RCT	72.0 (7.4)	40	24	60.00%	Inactive adults aged >60 years), moderate- high risk of CVD	PA + SB: Cognitive-behavioural counselling that focused on reducing SB and increasing light-intensity PA in addition to exercise to meet ACSM and AHA PA guidelines	20	Intervention completion	Exercise training for 8 weeks designed to meet ACSM exercise and PA guidelines for older adults
Rooijackers et al.	2021	Netherlands	Cluster RCT	82.1(6.94)	264	179	67.80%	Older adults ≥65 years old	SB: Behavioural intervention targeting home care staff	39 (9 months)	At week 52	Standard care
Rosenberg et al.	2020	America	RCT	68.4 (4.9)	36	25	69.40%	Older adults with obesity.	SB: I-STAND: Written materials, two in-person health coaching sessions of 30–60 min 1 week apart and four follow-up health coaching phone calls of 15–30 min over the remaining 10 weeks of the intervention.	12	Intervention completion	Educational material (healthy eating, stress management, and preventing falls)
Rosenberg et al.	2024	America	RCT	68.8 (6.2)	283	186	65.70%	Older adults	SB: I-STAND: Written materials, two in-person health coaching sessions of 30–60 min 1 week apart and eight follow-up health coaching phone calls of 15–30 over 6 months.	26 (6 months)	Intervention Completion	Health coaching – fall prevention, healthy eating, sleep
Suorsa et al.	2022	Finland	RCT	65.2 (1.1)	231	191	82.70%	Retirees	PA + SB: Activity Tracker: (i) daily activity goal and (ii) inactivity alerts.	52	Intervention completion	Waitlist Control
Tosi et al.	2021	Brazil	RCT	84.02 (6.52)	43	37	86.00%	Older adults with Frailty	PA + SB: Standing exercises, health education, and telephone support to reduce SB	16	Intervention completion	3 sessions educating harmful effects of SB
White et al.	2017	United Kingdom	Mixed-Method:Pilot RCT	68.32 (3.78)	103	57	55.30%	60 to 74 years, self- reportedly retired and sedentary (≥6 total leisure hours sitting per day)	PA + SB: Habit-based intervention: educational booklet, tips to increase PA and decrease SB, self-monitoring.	8	At week 12	UK government PA and suggestion that sedentary time is minimised.

Qualitative Articles	Year	Country	Design	Age (years)	Sample Size	Gender (F)		Population	Data Collection	Data Analysis
Aunger et al.	2020	United Kingdom	Mixed-Method Feasibility RCT	73.14 (5.78)	35	20	57.1%	Older adults >60 years oldListed for elective hip or knee surgery	Semi-structured interviews	Deductive realist thematic analysis
Bailey et al.	2024	United Kingdom	Mixed-Method: Feasibility RCT	74 (6)	60	40	67%	Older adults with very mild or mild frailty	Older adults with very mild or mild frailty	Semi-structured interviews
Blackburn et al.	2021	Denmark Germany Spain N. Ireland	Qualitative	76.9	38	17	45.00%	Older adults, took part in SITLESS trial	Semi-structured interviewsFocus groups	Thematic analysis
				72.6	27	13	48.00%			
				73.3	46	32	70.00%			
				74.3	39	23	59.00%			
Brookfield et al.	2015	United Kingdom	Qualitative	>60 years	22	16	72.7%	Healthy volunteers, stroke survivors and people with dementia	Semi-structured interviewsFocus groups	Inductive thematic analysis
Chang et al.	2022	Singapore	Qualitative	72 (5)	6	0	0.0%	Adults with COPD	Focus groups	Inductive ground-up
Chastin et al.	2014	United Kingdom	Qualitative	79.4 (7.75)	9	9	100%	Community-dwelling older adults	Semi-structured interviews	Framework analysis
Cheng et al.	2024	Australia	Qualitative	72.4 (9.21)	13	5	38.47%	Retired adults with moderate COPD	Semi-structured interviews	Deductive and inductive analysis
Collins et al.	2021	Canada	Qualitative	73.96 (NR)	27	19	70.4%	Community-dwelling older adults	Focus groups	Deductive thematic synthesis
Delobelle et al.	2025	Belgium	Mixed-method	72 (NR)	66	NR	NR	Community-dwelling older adults	Semi-structured interviews	Deductive analysis
Dontje et al.	2018	United Kingdom	Mixed-Method: Cross-Sectional	74 (5.3)	30	22	73.3%	Community-dwelling older adults	Semi-structured interviews	Inductive thematic analysis
Eklund et al.	2021	Sweden	Qualitative	70.36 (3.15)	14	8	57.1%	Retired community-dwelling older adults	Semi-structured interviews	Empirical phenomenological psychological (EPP) method,
Grady et al.	2021	United Kingdom	Qualitative	68.2 (6.6)	11	11	100%	People with Osteoporosis	Semi-structured interviews	Inductive and deductive thematic analysis
Greenwood-Hickman et al.	2016	America	Qualitative	71.7 (6.4)	24	16	66.7%	Overweight and obese older adults	Semi-structured interviews	Inductive thematic analysis
Hultman et al.	2024	Sweden	Qualitative	66.9 (2.9)	28	21	75%	Older adults transitioning to retirement	Focus groups	Qualitative content analysis
Leask et al.	2016	United Kingdom	Qualitative	78 (NR)	15	11	84.6%	Community-dwelling older adults	Focus groups	Thematic analysis
Leask et al.	2017	United Kingdom	Qualitative	74 (5.5)	11	6	54.5%	Community-dwelling older adults	Semi-structured workshops	Content analysis
Leitzelar et al.	2024	America	Mixed Method: Feasibility RCT	71.75 (8.4)	8	8	0	African American older adults	Focus groups	NR
Matei et al.	2015	United Kingdom	Mixed-Method: Uncontrolled trial	66.74 (4.34)	35	18	51.4%	Community-dwelling older adults	Semi-structured interviews	Thematic analysis
Matson et al.	2018	America	Qualitative	69.2 (4.9)	22	14	63.6%	Older adults with obesity	Semi-structured interviews	Inductive and deductive thematic analysis
McEwan et al.	2017	Canada	Qualitative	74 (8.5)	25	20	80.0%	Socially engaged older adults	Semi-structured interviewsFocus groups	Content analysis
McGowan et al.	2019	United Kingdom	Qualitative	76 (NR)	22	14	63.6%	Community-dwelling older adults	Semi-structured interviews	Inductive thematic analysis
McGowan et al.	2020	United Kingdom	Qualitative	77.5 (NR)	22	14	63.6%	Community-dwelling older adults	Semi-structured interviews	Inductive thematic analysis
McGowan et al.	2024	United Kingdom	Mixed-Method: Acceptability	74 (6)	15	11	73.3%	Older adults with sarcopenia	Semi-structured interviews	Framework analysis
Meghani et al.	2023	United Kingdom	Qualitative	72 (5)	49	21	43%	Older adults	Semi-structured interviews and focus groups	Thematic analysis
Meghani et al.	2025	United Kingdom	Qualitative	74 (6.4)	20	11	55%	Ethnically diverse older adults	Semi-structured interviews	Reflexive thematic analysis
Niklasson et al.	2023	Sweden	Qualitative	78.25 (6.24)	16	9	56%	Older adults	Semi-structured interviews	Phenomenological Hermeneutical
Niklasson et al.	2024	Sweden	Qualitative	78.25 (6.24)	14	6	43%	Older adults	Semi-structured interviews	Thematic analysis
Nuwere et al.	2022	America	Qualitative	75.6 (7.8)	46	41	89.1%	Community-dwelling older adults	Focus groups	Deductive thematic synthesis
Olanrewaju et al.	2022	United Kingdom	Randomised feasibility	74.6 (7.5)	11	6	45%	Community-dwelling adults aged 50+ years with mild cognitive impairment	Semi-structured interviews	Manifest content analysis
Orme et al.	2018	United Kingdom	Mixed-Method: Feasibility RCT	71 (20)	33	23	69.7%	Aged 40 to 85 years; confirmed COPD diagnosis, physically able to participate in light-intensity PA.	Semi-structured interviews	Thematic analysis
Palmer et al.	2018	United Kingdom	Qualitative	74.6 (NR)	44	21	47.7%	Community-dwelling older adults	Semi-structured interviews	Thematic analysis
Palmer et al.	2021	United Kingdom	Qualitative	74.6 (NR)	44	21	47.7%	Community-dwelling older adults	Semi-structured Interviews	Thematic analysis
Roderigues et al.	2023	Canada	Qualitative	72 (7.3)	21	13	62%	Pre-frail and frail older adults	Semi-structured interviews	Thematic content analysis
Tadrous et al.	2025a	United Kingdom	Qualitative	83 (0.89)	6	1	16.67%	Community-dwelling older adults	Focus groups	Inductive and deductive thematic analysis
Tadrous et al.	2025b	United Kingdom	Qualitative	83 (0.89)	6	1	16.67%	Community-dwelling older adults	Focus groups	Inductive and deductive thematic analysis
Tam-Seto et al.	2016	Canada	Qualitative	74 (8.5)	26	26	100%	Community-dwelling older adults	Focus groups	Directed content analysis
Trinh et al.	2015	Canada	Qualitative	73.5 (8.1)	27	0	0.0%	Prostate cancer survivors	Focus groups	Deductive thematic synthesis
UrrozGuerrero et al.	2024	Australia	Qualitative	67 (9)	21	13	62%	People with severe asthma	Semi-structured interviews	Inductive thematic analysis
Webber et al.	2019	Canada	Qualitative	67.5 (5.3)	22	14	63.6%	Adults with osteoarthritis and knee arthroplasty	Focus groups	Inductive thematic analysis
White et al.	2017	United Kingdom	Mixed-Method: Pilot RCT	68.32 (3.78)	103	57	55.3%	60 to 74 years, self- reportedly retired and sedentary (≥6 total leisure hours sitting/day)	Semi-structured interviews	Thematic analysis

### Quality appraisal

MMAT scores are reported in Table 2. Except for three studies [58, 66, 91], the remaining articles in the qualitative review scored 5/5 for their respective criterion. Three quantitative articles [46, 52, 54] scored 5/5, with the blinding of outcome assessors (13/21 studies) and participant adherence to the intervention (11/21 studies) being the lowest met criterion.

**Table 2:** Mixed-method appraisal tool assessment of included studies

Article		Year	Type	ScreeningQuestions	Quantitative Randomised					Qualitative					Mixed- Method					Quality
					1	2	3	4	5	1	2	3	4	5	1	2	3	4	5	
1	Barone Gibbs et al.	**2016**	RCT	✓✓	X	✓	✓	X	✓											3/5
2	Blair et al.	2021	RCT	✓✓	✓	✓	✓	X	X											3/5
3	Cheng et al.	2022	RCT	✓✓	✓	✓	✓	✓	X											4/5
4	Crombie et al.	2021	RCT	✓✓	✓	✓	✓	X	✓											3/5
5	English et al.	2016	RCT	✓✓	✓	✓	✓	✓	✓											5/5
6	Fanning et al.	2106	RCT	✓✓	X	✓	X	—	—											1/5
7	Kallings et al.	2009	RCT	✓✓	X	✓	—	—	—											1/5
8	Lyden et al.	2021	RCT	✓✓	✓	✓	✓	X	✓											4/5
9	Maher et al.	2017	RCT	✓✓	✓	—	✓	X	✓											3/5
10	Owari et al.	2019	RCT	✓✓	✓	✓	✓	✓	X											4/5
11	Roberts et al.	2019	RCT	✓✓	✓	✓	X	✓	✓											4/5
12	Rooijackers et al.	2021	RCT	✓✓	✓	✓	✓	—	X											3/5
13	Rosenberg et al.	2020	RCT	✓✓	✓	✓	X	✓	—											3/5
14	Rosenberg et al.	2024	RCT	✓✓	✓	✓	✓	✓	✓											5/5
15	Suorsa et al.	2022	RCT	✓✓	✓	✓	✓	✓	✓											5/5
16	Tosi et al.	2021	RCT	✓✓	✓	✓	—	✓	X											3/5
**17**	Blackburn et al.	2021	Qualitative	✓✓						✓	✓	✓	✓	✓						5//5
**18**	Brookfield et al.	2015	Qualitative	✓✓						✓	✓	✓	✓	✓						5//5
**19**	Chang et al.	2022	Qualitative	✓✓						✓	✓	✓	✓	✓						5//5
**20**	Cheng et al.	2024	Qualitative	✓✓						✓	✓	✓	✓	✓						5//5
**21**	Chastin et al.	2014	Qualitative	✓✓						✓	✓	✓	X	✓						4//5
**22**	Collins et al.	2021	Qualitative	✓✓						✓	✓	✓	✓	✓						5//5
**23**	Eklund et al.	2021	Qualitative	✓✓						✓	✓	✓	✓	✓						5//5
**24**	Grady et al.	2021	Qualitative	✓✓						✓	✓	✓	✓	✓						5//5
**25**	Greenwood-Hickman et al.	2016	Qualitative	✓✓						✓	✓	✓	✓	✓						5//5
**26**	Hultman et al.	2024	Qualitative	✓✓						✓	✓	✓	✓	✓						5//5
**27**	Leask et al.	2016	Qualitative	✓✓						✓	✓	✓	✓	✓						5//5
**28**	Leask et al.	2017	Qualitative	✓✓						✓	✓	✓	X	✓						4//5
**29**	Matson et al.	2018	Qualitative	✓✓						✓	✓	✓	✓	✓						5//5
**30**	McEwan et al.	2017	Qualitative	✓✓						✓	✓	✓	✓	✓						5//5
**31**	McGowan et al.	2019	Qualitative	✓✓						✓	✓	✓	✓	✓						5//5
**32**	McGowan et al.	2019	Qualitative	✓✓						✓	✓	✓	✓	✓						5//5
**33**	Meghani et al.	2023	Qualitative	✓✓						✓	✓	✓	✓	✓						5//5
**34**	Meghani et al.	2025	Qualitative	✓✓						✓	✓	✓	✓	✓						5//5
**35**	Niklasson et al.	2023	Qualitative	✓✓						✓	✓	✓	✓	✓						5//5
**36**	Niklasson et al.	2024	Qualitative	✓✓						✓	✓	✓	✓	✓						5//5
**37**	Nuwere et al.	2022	Qualitative	✓✓						✓	✓	✓	✓	✓						5//5
**38**	Palmer et al.	2018	Qualitative	✓✓						✓	✓	✓	✓	✓						5//5
**39**	Palmer et al.	2021	Qualitative	✓✓						✓	✓	✓	✓	✓						5//5
**40**	Roderigues et al.	2023	Qualitative	✓✓						✓	✓	✓	✓	✓						5//5
**41**	Tadrous et al.	2025a	Qualitative	✓✓						✓	✓	✓	✓	✓						5//5
**42**	Tadrous et al.	2025b	Qualitative	✓✓						✓	✓	✓	✓	✓						5//5
**43**	Tam-Seto et al.	2016	Qualitative	✓✓						✓	✓	✓	✓	✓						5//5
**44**	Trinh et al.	2015	Qualitative	✓✓						✓	✓	✓	✓	✓						5//5
**45**	UrrozGuerro et al.	2024	Qualitative	✓✓						✓	✓	✓	✓	✓						5/5
**46**	Webber et al.	2019	Qualitative	✓✓						✓	✓	✓	✓	✓						5//5
**47**	Aunger et al.	2020	Mixed-Method	✓✓	✓	✓	X	X	—	✓	✓	✓	✓	✓	✓	✓	✓	✓	—	11/15
**48**	Bailey et al.	2024	Mixed-Method	✓✓	✓	✓	✓	X	X	✓	✓	✓	✓	✓	✓	✓	✓	✓	—	13/15
**49**	Leitzelar et al.	2024	Mixed-Method	✓✓	—	X	—	—	✓	✓	✓	—	—	✓	✓	✓	X	X	X	7/15
**50**	Olanrewaju et al.	2022	Mixed-Method	✓✓	✓	—	—	X	✓	✓	✓	✓	✓	✓	✓	✓	✓	✓	—	11/15
**51**	Orme et al.	2018	Mixed-Method	✓✓	✓	—	X	X	X	✓	✓	✓	✓	✓	✓	✓	✓	✓	—	10/15
**52**	White et al.	2017	Mixed-Method	✓✓	✓	✓	X	X	X	✓	✓	✓	✓	✓	✓	✓	✓	✓	—	12/15
**53**	Delobelle et al.	2025	Mixed-Method	✓✓	**Quantitative Descriptive**					✓	✓	✓	✓	✓	✓	✓	✓	✓	—	12/15
					✓	—	✓	—	✓											
**54**	Dontje et al.	2018	Mixed-Method	✓✓	✓	—	✓	—	✓	✓	✓	✓	✓	✓	—	✓	✓	✓	—	12/15
**55**	McGowan et al.	2025	Mixed-Method	✓✓	✓	—	✓	—	✓	✓	✓	✓	✓	✓	—	✓	✓	✓	—	12/15
**56**	Matei et al.	2015	Mixed-Method	✓✓	**Quantitative non-randomised**					✓	✓	✓	✓	✓	✓	✓	✓	✓	—	12/15
					✓	**—**	✓	**—**	✓											

Shaded area denotes criteria not applicable

✓ Denotes criteria met

X Denotes Criteria not met

—Denotes unclear if criteria met

### Quantitative synthesis: interventions which aimed to reduce sedentary behaviour

**Fig. 2:**
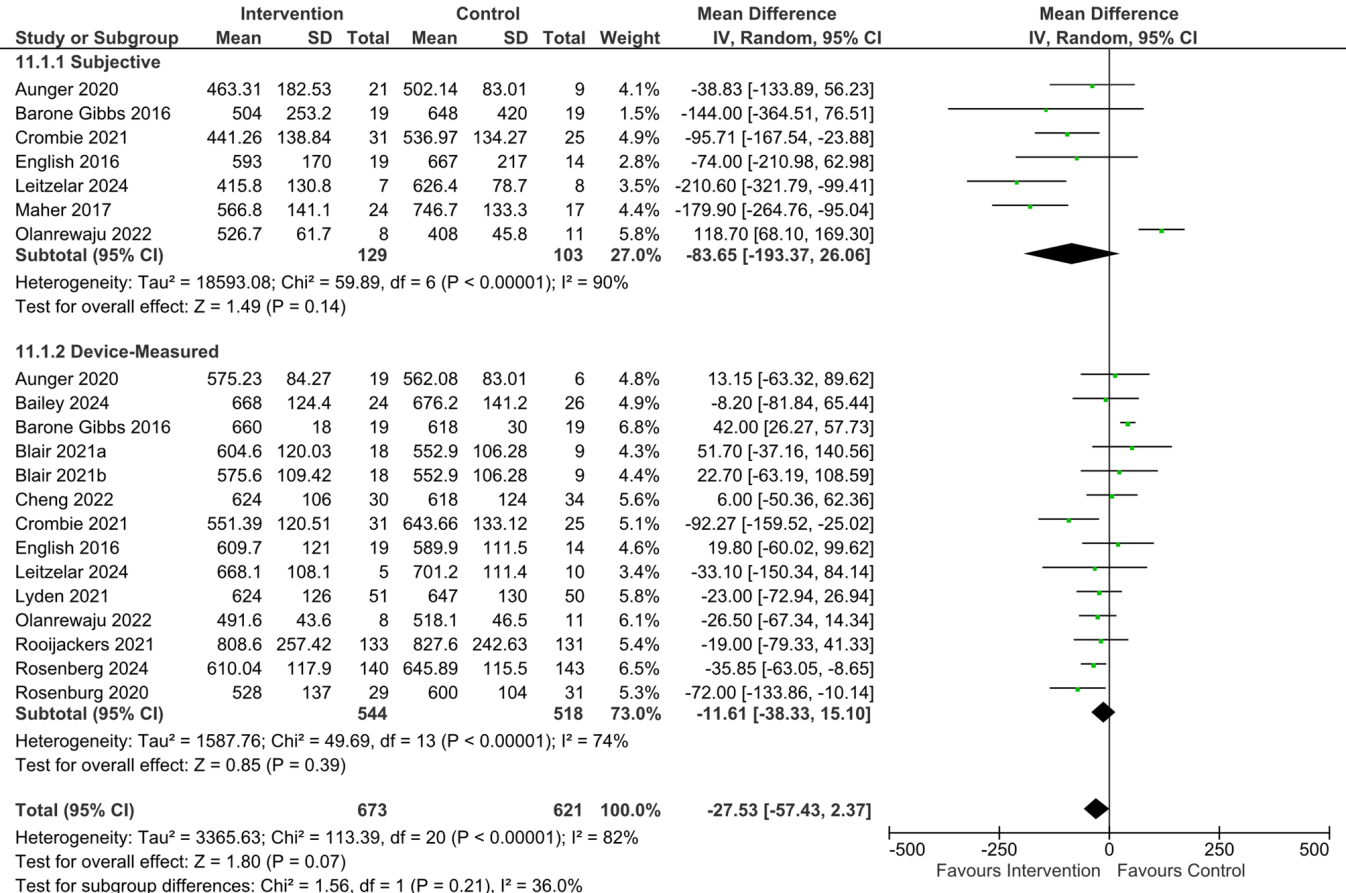
Forest plot of interventions to reduce sedentary behaviour vs. control

Interventions to reduce SB were pooled versus control (Fig. 2). Statistical heterogeneity was high for total sedentary time (I^2^ = 82%), the device-measured SB subgroup (I² = 74%), and the self-reported SB subgroup (I² = 90%). As such, a random-effects meta-analysis was performed. A total reduction of −27.53 min/day (95% CI −57.43 to 2.37, *P* = 0.07, I² =82%) was observed. A reduction of −11.61 min/day (95% CI −38.33 to 15.10, *P* = 0.39, I² =74%) was observed when device-based measures were pooled. A reduction of −83.65 min/day (95% CI −193.37 to 26.06, *P* = 0.14, I² = 90%) was observed when self-reported measures were pooled. There was an apparent mismatch between device-measured and self-reported SB, and when six studies [42, 45, 46, 85, 91, 92] that used both device-measured and self-reported SB were pooled, an additional 55.79-minute reduction in sedentary time was observed when self-reported (−64.68 min/day, 95% CI −181.50 to 52.14, *P* = 0.28, I² = 89%) compared to device-measured (−8.89 min/day, 95% CI −55.62 to 37.83, *P* = 0.71, I²= 79%). Age did not significantly moderate the effectiveness of interventions on reducing sedentary time, with older age showing a non-significant trend toward greater reductions (β = −0.0273, SE = 0.0327, *p* = 0.4029). The model explained none of the between-study variance (R² analog = 0.00). The effects of interventions on secondary outcomes are summarised in Table 3, with forest plots (including subgroup analyses according to intervention focus) and narrative summaries provided in the appendices.

**Table 3:** Effects of interventions on secondary outcome measures

Outcome Measure	Number of Studies; (Participants)	Mean Difference 95% CI	P Value	I^2^	References
Sit-to-Stand Transitions (n)	5 studies; n=438	−0.61 [−3.07 to 1.84]	0.62	14%	[43, 44, 47, 51, 55]
Sedentary Breaks (n)	5 studies; n=165	1.57 [−2.44 to 5.57]	0.44	37%	[45, 85, 86, 91, 92]
Sedentary Bouts Durations >30 mins	7 studies n=245	−6.15 [−29.78 to 17.48]	0.61	0%	[43, 44, 46, 85, 86, 92]
LIPA (mins/day)	6 studies; n=227	−10.97 [−25.76 to 3.81]	0.15	62%	[42–45, 91]
MVPA (mins/day)	8 studies; n=438	−1.04 [−11.48 to 9.40]	0.85	79%	[13, 14, 42–46, 91]
Steps	10 studies; n=842	550 [160 to 950]	0.006	0%	[13, 43, 44, 48, 49, 51, 52, 85, 86, 92]
Short Physical Performance Battery	8 studies; n=552	0.08 [−0.23 to 0.40]	0.84	26%	[15, 42, 43, 51, 53, 85, 86, 91]
Body Mass Index	7 studies; n=698	−0.67 [−1.25 to −0.09]	0.02*	0%	[13, 48, 49, 51, 52, 85, 86]
Waist Circumference (cm)	5 studies; n=580	−0.61 [−2.25 to 1.03]	0.46	0%	[15, 48, 49, 51, 52]
Systolic Blood Pressure (mmHg)	5 studies; n=512	−1.82 [−4.33 to 0.68]	0.15	65%	[15, 42, 48, 51, 52]
Diastolic Blood Pressure (mmHg)	5 studies, n=512	−0.14 [−2.84 to 2.56]	0.92	95%	

*Significant at p<0.05

### Intervention focus

The results of six interventions that aimed to increase physical activity and reduce sedentary behaviour [13–15, 47, 54, 85] were compared against fourteen studies that solely aimed to reduce sedentary behaviour [42–46, 49–53, 55, 86, 91, 92]. When device-based measures were pooled, interventions to reduce SB observed a slightly greater reduction (−11.61 min/day, 95% CI −38.33 to 15.10, *P* = 0.39) than interventions that aimed to increase PA and reduce SB (−10.78 min/day, 95% CI −34.20 to 12.65, *P* = 0.37, I² =95%). Similarly, when self-reported, interventions to reduce SB observed a greater reduction (−83.65 min/day, 95% CI −193.37 to 26.06, *P* = 0.14, I² = 90%) than interventions which aimed to increase PA and reduce SB (9.75 min/day, 95% CI −68.07, 87.57, *p* = 0.81).

### Intervention length

Interventions that aimed to reduce SB ranged from 2 to 36 weeks in length. Six interventions [44, 45, 50, 85, 91, 93] were classified as short-term interventions (≤ 6 weeks); four [42, 43, 46, 51, 92] were classified as medium-term interventions (7–16 weeks); and four [49, 52, 53, 86] were classified as long-term interventions (>16 weeks). When interventions that used device-based measures of SB were pooled, long-term interventions observed the greatest reductions (−29.12 min/day 95% CI −50.39 to −7.86, *P* = 0.007, I^2^ = 0%), followed by short-term interventions (−25.55 min/day − 78.51 to 27.40, *P* = 0.34, I^2^ = 50%); and medium-term interventions (4.70 min/day 95% CI −37.79 to 47.18, *P* = 0.83, I^2^ = 75%). As only three interventions to reduce SB included follow-up timepoints after intervention cessation [45, 53, 85], there is little indication if SB reductions are maintained.

### Intervention components

The BCTs of included interventions are reported in Table 4. Only three of 14 SB studies provided the BCTs incorporated in their interventions [43, 44, 85], with the rest coded. The most frequently included BCTs were as follows: Feedback on behavior (16/21), Goal setting (behavior) (16/21), Self-monitoring of behaviour (15/21), Action planning (13/21), and Adding objects to the environment (13/21). The effectiveness of the number of BCTs incorporated in 13 interventions to reduce SB that used device-based measures were examined [42–46, 49, 51–53, 85, 86, 91]. Interventions that incorporated more than 10 BCTs observed greater reductions of sedentary time (−24.01 min/day, 95% CI −46.90 to −1.11, *P* = 0.04, I² = 31%) than interventions which included 1–10 BCTs **(**9.24 min/day, 95% CI −31.41 to 49.89, *p* = 0.66, I^2^ = 67%).

**Table 4:** Behaviour change techniques (BCTs) of included studies

BCT			Aunger*	Bailey	Barone	Blair*	Cheng*	Crombie	English	Fanning	Kallings	Leitzelar	Lyden	Maher	Olanrewaju	Owari	Roberts	Rooijackers	Rosenberg 20	Rosenberg 24	Surosa	Tosi	White	# of BCTs
Goals and planning	**1.1**	Goal setting (behaviour)	✓	✓	✓	✓		✓	✓		✓	✓		✓	✓	✓		✓	✓	✓		✓	✓	**16**
	**1.2**	Barrier identification & problem solving	✓	✓	✓	✓	✓	✓				✓		✓	✓		✓		✓	✓		✓		**13**
	**1.3**	Goal setting (outcome)			✓		✓		✓	✓					✓			✓	✓	✓		✓	✓	**10**
	**1.4**	Action planning	✓	✓	✓		✓	✓	✓		✓	✓		✓	✓			✓				✓	✓	**13**
	**1.5**	Review of behavioral goals	✓		✓	✓	✓	✓				✓												**6**
	**1.6**	Review discrepancies					✓							✓										**2**
	**1.7**	Commitment									✓			✓										**2**
	**1.8**	Behavioral contract					✓																	**1**
Feedback and monitoring	**2.1**	Monitoring of behaviour by others w/o feedback			✓		✓																	**2**
	**2.2**	Feedback on behavior	✓	✓	✓	✓	✓		✓	✓		✓	✓		✓	✓	✓		✓	✓	✓	✓		**16**
	**2.3**	Self-monitoring of behavior	✓	✓	✓	✓	✓	✓		✓		✓	✓		✓				✓	✓	✓	✓	✓	**15**
Social support	**3.1**	Social support (unspecified)	✓	✓		✓		✓							✓			✓						**6**
	**3.2**	Social support (practical)	✓	✓		✓		✓	✓			✓					✓		✓	✓		✓		**10**
Shaping knowledge	**4.1**	Instructions on how to perform the behavior		✓		✓	✓	✓			✓				✓			✓	✓	✓		✓	✓	**11**
	**4.2**	Information about antecedents				✓									✓									**2**
Natural consequences	**5.1**	Information about health consequences	✓	✓		✓	✓			✓			✓	✓	✓	✓					✓	✓	✓	**12**
Comparison of behaviour	**6.1**	Demonstration of the behaviour					✓			✓		✓		✓									✓	**5**
Associations	**7.1**	Prompts/cues	✓	✓		✓	✓	✓		✓				✓					✓	✓	✓	✓	✓	**12**
Repetition and substitution	**8.1**	Behavioral practice					✓											✓						**2**
	**8.2**	Behaviour substitution	✓				✓									✓							✓	**4**
	**8.3**	Habit formation														✓		✓	✓	✓			✓	**5**
	**8.4**	Habit reversal																					✓	**1**
	**8.6**	Generalization of a target behavior				✓																		**1**
	**8.7**	Graded tasks				✓	✓	✓		✓	✓	✓										✓	✓	**8**
Comparison of outcomes	**9.1**	Credible source							✓		✓	✓		✓					✓	✓		✓		**7**
Antecedents	**12.1**	Restructuring the physical environment	✓				✓																✓	**3**
	**12.3**	Avoidance/reducing exposure to cues					✓																	**1**
	**12.5**	Adding objects to the environment		✓	✓			✓		✓			✓		✓	✓	✓	✓	✓	✓		✓	✓	**13**
Identity	**13.2**	Framing/reframing					✓					✓											✓	**3**
	**13.3**	Incompatible beliefs					✓																	**1**
Self-belief	**15.1**	Verbal persuasion re capability					✓											✓						**2**
Number of BCTs per study *= BCTs provided by study			**12**	**11**	**9**	**13**	**21**	**11**	**6**	**8**	**6**	**11**	**4**	**9**	**11**	**6**	**4**	**9**	**11**	**11**	**4**	**13**	**15**	**205**

### Publication bias

**Fig. 3:**
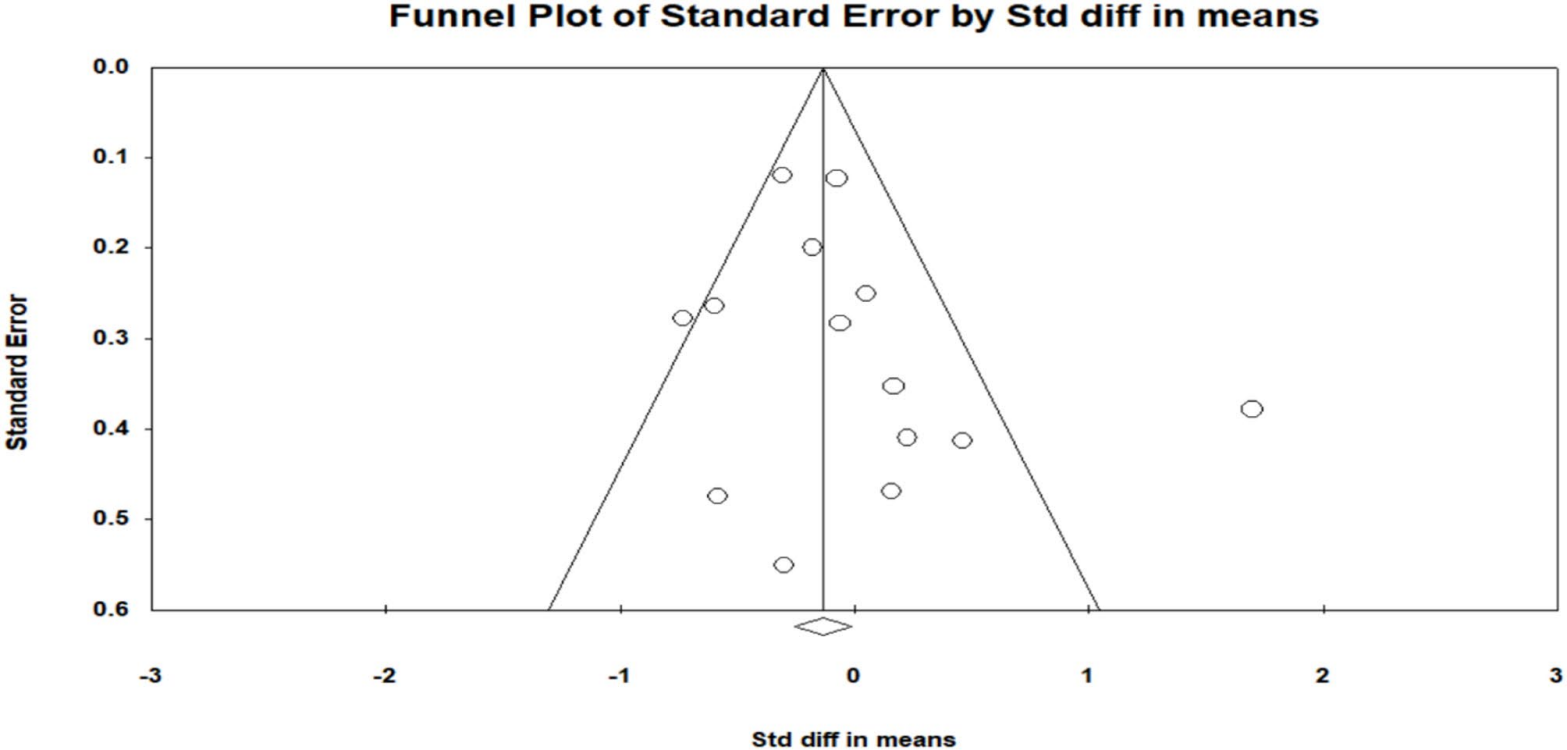
Funnel plot of standard error by standardised difference in means

The funnel pot (Fig. 3) appeared largely symmetrical, and Egger’s regression test indicated no significant asymmetry (p=0.29). Additionally, lDuval and Tweedie’s trim and fill analysis did not identify any missing studies, with adjusted effect sizes remaining unchanged. These findings suggest a low risk of publication bias in the included studies.

#### Qualitative synthesis

##### Activities performed in sitting

The activities performed by older adults were extracted [58, 59, 61, 63–65, 67, 68, 70–75, 77, 78, 80–84, 88, 92, 93] and charted to the relevant domains of the ecological model of SB [94] (Table 5). Sitting activities were predominantly leisure-related and occurred in older adults’ homes. Palmer et al. [76] reports that sedentary activities did not vary according to SB levels, but individuals with higher SB would perform sedentary activities more frequently or for longer.

**Table 5:** Activities performed in sitting and standing

Activities in Sitting	Activities in Standing
Leisure-Related Sedentary Behaviour	
• TV, Reading • Puzzles/Crosswords • Tablet/Computer • Resting/Relaxing • Knitting/Sewing • Playing games/cards • Socialising	• Walking • Shopping • Exercise, dancing • Gardening • Community classes • Socialising • Going to museums/libraries
Transport-Related Sedentary Behaviour	
• Bus • Train • Driving	• Walking • Walking to/from public transport • Cycling
Occupation-Related Sedentary Behaviour	
• Employment requires sitting • Computer usage • Teaching • Attending courses	• Volunteering • Carer role (family members/spouse) • Helping friends
Household-Related Sedentary Behaviour	
• Eating • Sorting medication • Computer usage • Resting/Napping	• Preparing meals • Answering phone calls • Getting dressed • Chores/tidying up • Getting medication • DIY

### Barriers and facilitators to reducing sedentary behaviour

The barriers and facilitators to reducing SB as expressed by participants were charted against the COM-B model of behaviour (Table 6) and discussed narratively below:

**Table 6:** Barriers and facilitators charted to COM-B model of behaviour

Physical Capability	Pain	“It was very painful to walk and so I got...to be very sedentary probably because it hurt to move.”	[63]	Reduce Pain	“If I’m not moving, it puts a little stress on my back and makes my back sore, so I’m trying to find more opportunities for standing.”	[67]
	Fatigue	“It is difficult to maintain the healthy behaviour on your own, especially at our age, you need a lot of energy to get going, and I must admit that it’s getting more and more difficult.”	[56]	Reduce Stiffness	“You’re immediately rewarded when you stand up and you’re not so stiff that you can’t walk.”	[63]
	Physical Health Problems	“It’s a case of...the spirit is willing, but the body is weak. The thought of actually getting up and going out and things like that, you just know how the pain is going to be, how tedious it is, getting about and things, so forget it"	[69]	Manage Physical Health	“Significantly better well-being, I want to keep that as long as possible.” (referring to effect of intervention)	[56]
	Chronic Health Problems	“I knew I needed to start moving more, without a doubt, and I – I have arthritis in my knees and my foot and my shoulders and my back, and it’s really easy to sit down and do nothing”	[63]	Increase Energy	“So, I enjoy walking outside. And that made me feel stronger. Yes, that made it easier to stay up more”.	[63]
Psychological Capability	Depression	“When you’re in my situation, you are terribly lonely and as a consequence the easiest thing...didn’t say it was the right thing, the easiest thing to do is to sit and watch that thing [television] or put your feet up”	[69]	Improved Sleep	“When I do the exercises, I feel a bit tired, and then by the evening... I go to sleep. I do sleep well. Before I couldn’t [sleep], I used to twist and turn, had aches and pains... but [since] then it’s improved.”	[89]
		“If I’ve not like gone out for say three or four days I actually get more depressed and ill... I feel stronger doing stuff’	[70]	Improve Focus	“Like when I clean the house, I didn’t get nearly as exhausted as I have in the past.”	[63]
	Anxiety	“For me one of the...the big things was after spending a few years basically isolated, was the fear of coming, of not fitting in, of... you know, not belonging, um... It was very scary for me to come here...”	[81]	Improved Satisfaction	“I think it would be good for it [mood] yes, yeah. Because if you’re sitting down, you have more time to think and you’re brooding.”	[70]
	Knowledge	“I don’t think people know [about health risks and sedentary behaviour]. I never knew about them until I came here.”	[84]	Improve Mental Wellbeing	“If I wasn’t enjoying it, I wouldn’t do it, you know I’d come back home and watch telly... It’s the enjoyment that would keep me going”	[69]
	Reduced Confidence	“Well, I think people go into a rut and lose their confidence about just going out the front door.”	[69]			
Physical Opportunity	Lack of Time	“But lots of time I get up in the morning and I find that I don’t have enough hours in the day to do everything that I want to get done... and I think that I’m really busy, I think I’m really doing something, you know?”	[84]	Employment/Volunteering	“I think that the people that are volunteers at, OSCC are a different, different group of people all together than you’re gonna find in a retirement home or something like that because we’re busy volunteering, we’re doing things we’re a different breed of people than anything else.”	[81]
	Lack of Space	“This flat’s too small to do things in, it’s too small. Nothing in my house I can do really is it [there]. Can’t do much in your flat can yer [you].”	[69]			
	Lack of Activities	“If I’ve got somewhere to go I’ll go. But I’ve nowhere to go, what can I do? I got nothing to do so I watch television…nobody offers anything around here.”	[69]	Interesting Activities Available	“They’re getting outta bed, they have to get dressed, they have to either you know, um... Walk or take a bus or get a taxi or get a ride or something. They gotta walk through the parking lot and walk in, you know.... We’ve had people huffing and puffing by the time they get in here with their walkers, but I mean, that’s, that’s already more exercise than they would have had sitting at home.”	[68]
	Environmental Influences	“My husband and I would have come down [a local walk] with the dogs and we loved that but now hills and things really restrict me. More recently as I’m now on my own, I’m even more restricted due my health and no longer driving has an impact on how much I get out and about.”	[56]			
	Bad Weather	“I think it’s also seasonal because, basically in the wintertime, there’s a tendency [for] the human to kind of hibernate, sit down more inside—you can’t go out and do a lot more. As the weather gets right, everybody wants to get outside... and enjoy the weather."	[82]	Indoor Activities	“During the bad weather I usually wouldn’t get out but having this centre has meant that I can keep it up without having to be outside walking.”	[56]
	Financial Constraints	“And do you want me to tell you another thing? Money. It costs a lot to take part in almost everything here.”	[81]	Home Enabling Reduction of Sedentary Behaviour	“Having a two-story house helps. I probably do eight flights of stairs a minimum a day. I teach on one floor and live on the other.”	[63]
	Poor Public Transport	“You’ve got to drive to [nearby town] or that to get to the swimming or the gym or whatever, and you think, God, if it was only nearer you could maybe walk part of the way or whatever but, no, there’s not a great amount of things locally that you could go to.”	[76]		“I mean I do live in a house so I do have to go upstairs...When you potter round a house there’s always something you can see to do really, you know what I mean”	[69]
Social Opportunity	Loss of Role	“Well, since retiring. I would’ve said. And I retired at 60. I had a job where I was on their feet for the full eight hours of work and on an evening when the telly goes on, I’ll sit, sit read all evening and not move.”	[80]	Social Support: Family	“Son and his family up... I never sat down because they were all around the house moving about”	[65]
	Social Isolation	“I lived here 14 years, and I can honestly say, I’ve no friends, I’ve nowhere to go...So I’ve nothing to do so I just sit about.”	[76]		“Well, my daughter said to me ‘you’re going to keep it up, aren’t you?’ and of course I am but she was really glad that we were doing it.”	[56]
	Social Expectation	“You always have the kids who... say you shouldn’t be doing that mom. You know, you shouldn’t be lifting that... or you might hurt yourself. Yeah, careful you’re going to fall.”	[84]	Social Support: Friends	“Well, I had a bunch of gals up here at the park that I walk with. They all thought it was a hell of a good idea. They were real proud of me”	[63]
	Reduced Social Support Network	“It’s awful if you’ve got to go on your own... so they shut the door and stay in.”	[69]		"If you were going out just for the evening, yeah, for to do whatever, you know, the theatre or just meet friends. Because you wouldn’t be sitting"	[80]
Reflective Motivation	Not Aware of Consequences of Sedentary Behaviour	“I’m a fairly active person, even though I guess according to the charts, I’m not. But as far as I and my friends are concerned, I’m pretty active...You have to convince me that the way I spend my life sitting and standing is unhealthy.”	[63]	Maintain Independence	“Then it is a motivation I have to keep doing what I learned. Because if it does good to me, why stop? Anyway, it is no effort...”	[56]
	Unaware of Sedentary Behaviour	“I was surprised that I didn’t stand more, because in my head I’m standing more. And my friends all think of me as somebody that’s always on the go doing stuff, so I was quite surprised that I didn’t have more standing in there, you know.”	[77]	Awareness of Health Benefits	“I think when you sit a long time you get stiff. I think it’s much better to be active and move about because if you’ve got an ache or a pain and you walk about, it goes away whereas the longer you sit, the stiffer you get”	[76]
	Lack of Confidence	“Well, I think people go into a rut and lose their confidence about just going out the front door.”	[69]	Control over Health	“I can follow this programme and keep myself as mobile or as physically able as possible. It gives me hope and encouragement, and that makes me happy.”	[89]
	Fear of Falling	“You don’t wanna slip and fall and hurt yourself. It’s not that easy to get up again, you know”	[68]			
Automatic Motivation	Habitually Sedentary	“It’s also very easy to slip back into old patterns. I mean, in our 70 s, we have pretty ingrained patterns.”	[63]	Habitually Non-Sedentary	“And I had a job with the engineering where I didn’t sit down from going in to coming out. It was active all the time. Now it’s gardening, cycling or walking.”	[80]
	Enjoy Activities in Sitting	“Things like reading a book or doing your knitting - getting up, that wouldn’t be fun, it would spoil the enjoyment.”	[80]		“I worked as a living, that I was out every day and I had an elderly parent I looked after...I would not have liked to have to be staying in all the time.”	[76]
	Sitting as Rest/Reward	“I was looking forward to this aspect of retirement that I, you know—I’d be able to indulge some of my other passions like reading, studying, intellectual pursuits... And I kind of resented this idea of: What do you mean, I can’t sit down?”	[67]	Activities of Daily Living	"I do find myself moving around quite a bit for chores round the house."	[88]
					“Well, some days the spirit moves me, and I’ll clean the house”	[65]
		“You need so much energy to get through the day, it’s difficult when you get home, and you’re trying to recover and getting up is sort of difficult then, you just want to sit and relax and get better.”	[93]		“I love my gardening so that takes care of the spring time when you have to dig up and, and, and, and clean up your garden and plant new plants and stuff and then cutting the grass every week.”	[81]

### Physical capability

Older adults reported that performing activities in standing was difficult due to physical health problems such as pain, fatigue, and stiffness [56, 58, 63, 65, 67, 68, 70, 77, 80–84, 88, 92, 93]. Sitting was viewed as a means to reduce pain and stiffness and manage fatigue [58, 61, 63–65, 67, 68, 70, 73–75, 77, 81, 82, 84, 88, 90, 93]. Some older adults recognised that their sitting contributed to their pain and stiffness and that interrupting their sitting was an important way to reduce pain and stiffness and manage their physical health and fatigue [56, 58, 63, 80, 82].

### Psychological capability

Feelings of depression, anxiety, and reduced self-esteem promoted SB [58, 69, 70, 77, 80, 81]. Contributing factors included the loss of role following retirement [69, 70, 77, 81], the bereavement of a spouse/friend [68–70, 77, 81], or fear of falling [57, 68, 69, 81, 84]. Older adults were motivated to reduce their SB because of the beneficial effects on their mental well-being, such as improved sleep, mood, and confidence [14, 63, 67, 80, 90].

#### Physical opportunity

Barriers included a lack of space or stairs within the home [63, 69, 80, 84, 92], limited public seating and recreational facilities [69, 77], poor public transport [56, 58, 68, 69, 77, 81, 84, 88], financial constraints [69, 81], poor weather [56, 58, 63, 68, 69, 77, 80–84, 90], community safety and anti-social behaviour [69]. Facilitators included employment, volunteering, and interesting and appropriate activities available for older adults to engage in [56, 68, 69, 88, 90]. The home also facilitated the reduction of SB, with features like stairs or gardens encouraging the reduction of sitting time [57, 58, 63, 64, 67, 69, 71, 80].

#### Social opportunity

Older adults described a social expectation for them to be sedentary, with carers, family, and friends limiting their activities by encouraging sitting [58, 63, 81, 84]. The loss of role following retirement, accompanied by reduced organisation or structure to their day, also promoted SB [14, 64, 69, 80]. Reduced social support discourages older adults from undertaking activities, and social norms to sit whilst working or eating further promote SB [63, 65, 67, 68, 77, 84, 89]. Facilitators included employment, which provided a sense of purpose, visits from family or friends, and social support to reduce their SB [56, 58, 59, 63–65, 67–69, 71, 77, 80–84, 88–90].

### Reflective motivation

Older adults were frequently unaware of their SB, the detrimental effects of their sitting, or the benefits of reducing their SB [56, 59, 63–65, 68, 70, 77, 79, 80, 82, 84, 90]. Fears of mobilising or falling further reduced confidence in performing non-sedentary activities and increased home-based SB [57, 58, 68, 69, 81, 84]. Some older adults were aware of the benefits of reducing their SB in managing their well-being and maintaining functional independence [63–65, 70, 77, 80, 82].

### Automatic motivation

Barriers included older adults being habitually sedentary [58, 63, 67–69, 77, 82, 84, 88], enjoying activities in sitting [59, 67, 68, 82, 88, 90], and sedentary activities being easier to perform than activities in standing [69]. Older adults often reported viewing sitting as a rest or reward after a hard day’s work [58, 63, 67–70, 77, 84, 93]. Conversely, some older adults reported being habitually non-sedentary [58, 70, 76, 80] and having activities of daily living (ADLs), such as preparing meals, taking medication, that were performed in standing which facilitated reducing their SB [58, 66–69, 77, 80–82, 84, 88, 93].

### Intervention effects and desired components

The findings of 12 studies that explored older adults’ experiences with interventions to reduce SB were synthesised [14, 56, 59, 63, 71, 85–87, 90–93] and are summarised in Table 7. Reported effects of interventions included increased knowledge and awareness of their sedentary behaviour [14, 56, 59, 63, 71, 85–87, 90, 93], behaviour change and habit formation [14, 59, 63, 71, 86, 87, 90, 92], psychological benefits such as improved mood and wellbeing [63, 71, 85, 86, 90], and physical health benefits such as improved strength, energy, sleep and walking ability [14, 56, 59, 63, 71, 85, 86, 90]. Responses relating to older adults’ perception of intervention components were extracted and grouped according to the appropriate BCT domain [14, 56, 59, 63–66, 69–71, 79, 82, 85–87, 89–93].

**Table 7:** Reported effects of interventions and desired intervention components

Reported Effects of Interventions	Desired Aspects of Interventions
**Awareness and Motivation**	**Goals and Planning**
• Increased awareness of SB	• Goal setting and follow up
• Increased motivation to reduce SB	• Health coaching
**Behavioural Change**	**Feedback and Monitoring**
• Reduced sedentary behaviour	• Self-monitoring of behaviour
• Performing more activities in standing	• Device-based monitoring
• Increased physical activity (PA) levels	• Feedback on behaviour
• Habit formation and lifestyle change	
**Physical Outcomes**	**Social Support**
• Reduced pain and stiffness	• Group sessions
• Weight loss	• Peer support
• Improved strength and balance	• Family/friend support
• Improved walking and mobility	• Health coaching
• Improved ability to perform daily activities	**Shaping Knowledge**
• Improved energy levels	• Educational sessions
• Improved sleep quality	• Educational booklet/pamphlet
	• Instructions on how to perform behaviour
**Psychological Outcomes**	**Associations**
• Improved mood	• Prompting/Cueing
• Reduced stress	• Technological prompts
• Greater sense of control	• Environmental restructuring
**Social & Broader Impact**	**Comparison of Outcomes**
• Increased social participation	• Receiving information from credible source
• Improved overall health and wellbeing	
• Sharing knowledge with others	

### Analytical theme 1: what sitting means to older adults

Although SB can be defined, what sitting means to older adults is highly individual. To some, sitting is synonymous with self-management to remedy pain, fatigue, or physical health problems [58, 61, 63–65, 67, 68, 70, 73–75, 77, 81, 82, 84, 88, 90, 93]. To others, sitting provides a sense of safety for those afraid to leave the home [69] or falling [57], or those adapting to the loss of role following retirement [8]. Sitting can be a means for social interaction [63], a reward after a hard day’s work [67], or an opportunity to complete activities to stave off cognitive decline [76]. Older adults have also described sitting as being “vegetating” or “stagnating” [70], “lacking discipline” [84], or being “better off dead” [70]. Additionally, participants often conflated SB with being physically inactive, perceiving the two behaviours as opposing points along a single behavioural spectrum [70, 84]. When prompted about how they could reduce their sitting time, older adults would frequently propose strategies to increase their PA [70], indicating a tendency to frame behaviour change through movement rather than reduction. Many older adults did not appear to fully recognise the health risks associated with SB, nor the health benefits associated with reducing their sedentary time. Even those aware of SB and the associated negative health consequences reported excessive SB, which suggests that education alone may not be sufficient to reduce SB in this population.

### Analytical theme 2: expectations of ageing

Ageing was frequently linked with an expected deterioration in physical capability [57, 68, 80, 83], with many experiencing problems such as pain, fatigue, and stiffness [56, 58, 63, 65, 67, 68, 70, 77, 81–84, 88, 92, 93]. These complaints were frequently remedied by sitting [58, 61, 63, 65, 67, 68, 70, 73–75, 77, 81, 82, 84, 88, 93]. However, few recognised that their SB may contribute to their physical health problems [63, 70, 76, 82, 95]. This was exacerbated by a reduced capability to undertake activities involving prolonged standing [58, 62, 68, 69, 72–74, 77, 81]. The loss of a structured routine post-retirement was cited as a factor that encouraged sedentary habits in later life [61, 64, 69, 76, 80, 81]. Retirement presents older adults with the time and freedom to do what they enjoy, which frequently involved sedentary activities [61, 68, 89]. Additionally, the language used to describe older adults, such as ‘senior citizens’, was perceived as stigmatising, reducing their willingness to participate in activities [68]. There was a sentiment that the changes associated with ageing should be acknowledged, but that ageing was not synonymous with decline [69, 81].

### Analytical theme 3: social influences in older adults

Social influences can be both beneficial and detrimental to reducing SB. Family and friends can inadvertently limit the activities of older adults out of respect, social or cultural norms, or fear that the older adult may injure themselves [58, 63, 81, 84]. This limitation of activity can reduce older adults’ independence and activity by promoting cautious avoidance [58]. Furthermore, bereavements and diminishing social connections often led to withdrawal from formerly valued activities, contributing to increased sedentary time [68–70, 77, 80, 81]. When combined with the loss of role following retirement, older adults can often feel socially neglected and report having few reasons to leave their homes [61, 68–70, 77, 81]. Social influence can also be beneficial in reducing SB. Social engagement through caring for grandchildren or visits from family or friends was seen as a motivating factor in reducing sedentary time [56, 58, 62, 63, 66, 72, 77, 88]. Volunteering also provided social enrichment and structure to mitigate the loss of role following retirement [56, 68, 69, 88]. Other positive influences included loved ones following up or supporting older adults to reduce SB [59, 64, 67–69, 71, 77, 81–84, 89, 90].

## Discussion

This review aimed to synthesise quantitative and qualitative studies to explore the suitability of interventions to reduce SB in community-dwelling older adults. Quantitative and qualitative findings will now be narratively integrated to identify complementarity and/or discrepancy present regarding the study populations, outcome measurement, and intervention design (Table 8).

**Table 8:** Overview of quantitative and qualitative findings and review recommendations

	Quantitative	Qualitative	Review Recommendations/Findings
Study Population	- Predominantly white, female (66.49% female; n=1288). - Aged 65–74 (71.62 ± 8.57 years) - Western countries	- Predominantly white, female (56.78%; n=611). - Aged 65–74 (72.48 ± 7.19 years) - Western countries	- Adults aged ≥75 years underrepresented. - Predominantly Western countries - cannot identify cultural differences. - More inclusive recruitment strategies, recruit underrepresented subsets of older adults. Target adults ≥ 75 years.
Outcome Measurement	- All included measure of Sedentary Behaviour. - 3/21 RCTs measured time in sedentary activities. - Potential self-report bias with subjective measurements of SB. - Secondary outcome measures underutilised (e.g. 6MWT used in 3 studies vs step counts used in 11 studies.	- Not interested in reducing SB solely to reduce their sedentary time. - Value tangible health benefits associated with reducing SB such as reduced pain, stiffness, improved HRQoL and sleep. - Need convincing that their sitting time is detrimental, and that reducing SB would improve their health.	- Mismatch between research and older adults’ priorities present. - Older adults valued receiving information about health benefits associated with reducing SB and consequences of prolonged SB from credible sources as intervention components. - To do so, interventions must include secondary outcome measures that measure effects of SB on outcomes valued by older adults e.g. pain, stiffness, to further develop the evidence-base. - Combination of device-measured (accuracy) and self-reported measurements (context) of SB recommended.
Intervention Duration	- Evenly distributed intervention lengths - Long-term interventions (>16 weeks) were more effective (−29.12 mins/day) than medium-duration (7–16 weeks) interventions (+4.70 mins/day) and short-term interventions (>16 weeks) (−25.55 mins/day) - Follow-up periods infrequently incorporated (2/14 sedentary behaviour interventions)	- Concerned re habitual nature of sedentary behaviour and short durations of interventions. - Feel original behaviours may return upon cessation of interventions. - Follow-ups may promote intervention adherence.	- Long-term interventions (>16 weeks) appear to be most effective currently. - Need to incorporate follow-up periods in interventions to explore maintenance of behaviour following intervention cessation
Intervention Level	- 20/21RCTs targeted individual level - 1/21 targeted provider-level - No system-level interventions	- Expressed interest in system-level and provider-level interventions. - **System: **Described how the home and their communities contribute to increased sedentary time. - **Provider:** Value education from healthcare professionals, unaware of sedentary time, poor understanding of sedentary behaviour and its’ effects.	- Explore system-level and provider-level interventions: - **Provider-level:** would target healthcare providers interacting with older adults. Focus on education and screening to identify sedentary individuals. - **System-level: **Improving local services and facilities (e.g. public transport, community services, activities, public seating) and home to reduce SB.
Intervention Content	- Most incorporated BCTs: Feedback on behavior (16/21), Goal setting (behavior) (16/21), Self-monitoring of behaviour (15/21); Action planning (13/21) and Adding objects to the environment (13/21). - Interventions to reduce SB slightly more effective than interventions which aimed to increase PA and reduce SB (−11.62 mins/day vs −10.78 mins/day) - Using ≥10 BCTs more effective than 1–10 BCTS (−24.01 mins/day vs −9.24 mins/day)	- Most desired BCTs: self-monitoring, social support, information about health consequences, prompting/cueing. - Technological interventions divisive. - **Pros:** provides information re sedentary bouts and objective sedentary time - **Cons**: bulky, irritating, uncomfortable, attracts attention	- Slight mismatch re intervention components. Social support was the most requested yet only used in half of the included RCTS. - More accurate reporting of intervention components necessary 3/14 SB interventions provided BCTs). - Incorporate older adults’ preferences in intervention content.

### Study population

With both syntheses predominantly recruiting white females, the transferability of findings to wider populations of older adults is limited, particularly with minority ethnic communities. Similarly, the population of older adults aged ≥ 75 years is considerably underrepresented. Of the 56 articles included, only nine qualitative studies [58, 66, 69, 70, 73–75, 79, 80] and three RCTs [50, 53, 55] recruited samples with a mean age of ≥ 75 years. Consequently, our understanding of SB and the appropriateness of existing interventions has predominantly been informed by a younger subset of older adults.

Progression through older adulthood is oftentimes accompanied by a reduction in functional ability, with Age UK reporting that the percentage of people experiencing difficulties with their ADLs increases from 15% in those aged 65–69 to 1-in-3 people requiring some level of care and support by age 85 [96]. Ageing is also associated with reduced social support networks and social isolation, which can lead to increased sedentary behaviour [97] and poorer physical and mental wellbeing [98], as highlighted by the thematic synthesis. Given these challenges, and the increased frailty [99], balance impairments [100], and cognitive decline present [101], tailored strategies to reducing sedentary behaviour in this subsection of older adults may be warranted. Future research should explore the effects of interventions to reduce SB in adults aged ≥ 75 years, as this population is expected to double over the next 30 years [102]. Broader recruitment strategies should also be employed to ensure underrepresented communities are recruited.

### Outcome measurement

Considerable mismatches exist between self-reported and device-based measurements of SB. When measured subjectively, an additional 72.03-minute reduction is observed. When six interventions that employed both subjective and device-based measures of SB were pooled [42, 45, 46, 85, 91, 92], an additional 55.79-minute reduction in SB was observed. Despite this difference not being statistically significant (*p* = 0.38), this mismatched reduction of SB determines if the minimal-clinically important difference (MCID) is reached, with reductions of 30–60 min/day associated with improvements in cardiometabolic health, HRQoL, and reduced mortality [103–105]. Relying solely on self-reported measures of SB may therefore overestimate intervention effectiveness and distort the perceived impact on secondary outcomes. Self-report bias must also be considered when subjectively measuring SB in older adults, as an international consensus statement on SB in older adults reported that self-reported measures of SB have limited validity and reliability [106]. However, self-report measures can contextualise sedentary activities (e.g., reading or watching television). Future research should incorporate device-based and self-reported measurements of SB to accurately measure SB and contextualise sedentary activities, respectively.

Beyond measurement discrepancies, a further mismatch was observed between the outcome measures used in existing interventions and the outcomes that older adults valued. Qualitatively, older adults frequently expressed that they did not want to reduce their sitting time for the sole purpose of reducing their SB, and that interventions must be meaningful [56, 63, 64, 69, 70]. This may partly reflect that some sedentary activities such as socialising, computer-use or reading, are mentally engaging and can be beneficial [107]. Furthermore, few interventions investigated secondary outcomes as highlighted in the quantitative review. From the mixed-method studies, older adults valued the tangible benefits they observed from participating in interventions such as reduced pain and stiffness [14, 56, 63, 67, 89], and receiving education from credible sources, such as healthcare professionals [66, 67]. However, the omission of wider outcome measures limits the evidence base by which we can educate older adults about the health benefits of reducing their SB. For example, only six RCTS included HRQoL measures [42, 43, 45, 85, 91, 92]. With cross-sectional studies suggesting an inverse relationship between sedentary time and HRQoL, particularly among the oldest old [108], future research should incorporate measures of HRQoL to determine if reducing sedentary time can improve HRQoL in this population.

### Intervention efficacy

When compared to the review by Chastin et al. [11], we observed a smaller reduction of total sedentary time (−27.53 min/day, 95% CI −57.43 to 2.37, *P* = 0.07, I² =82% vs. −44.91 min/day, 95% CI −93.13 to 3.32, *P* = 0.58; I^2^ = 73%) although our results offer greater precision. We observed similar reductions in self-reported SB (−83.65 min/day, 95% CI −193.37 to 26.06, *P* = 0.14, I² = 90% vs. −84.29 min/day; 95% CI −270.14 to 101.56, *P* = 0.001, I^2^ = 90%). The smaller reductions in device-measured sedentary (−11.61 min/day 95% CI −38.33 to 15.10, *P* = 0.39 vs. − 30.45 min/day; 95% CI – 72.68, 11.77; *P* = 0.06; I^2^ = 57%) may be attributable exclusion of interventions conducted in clinical populations in the review by Chastin et al. [11], as we included studies in populations such as those awaiting surgeries [85], cancer survivors [43], stroke survivors [46], chronic kidney disease [49] and chronic obstructive pulmonary disease [44]. The lower reduction of device-based SB may also be attributable to the lower age of inclusion in the review by Chastin et al. [11], as studies were predominantly conducted in western countries where retirement commonly occurs at ≥ 65 years. As the qualitative review suggests, retirement contributes considerably to SB due to the associated loss of role, organisation, and structure. Although the mean participant age for certain studies included by Chastin et al. [11] was ≥ 60 years, the eligibility criteria for the individual studies, and as such, the resulting sample may capture adults below this threshold and inflate the effectiveness of included interventions.

### Intervention level

Interventions typically operated at an individual-level, except for the provider-level intervention by Rooijackers et al. [53]. From the qualitative synthesis, older adults expressed how system-and-provider-level interventions could act. System-level interventions could restructure the home and communities to promote the reduction of SB [109]. Additionally, providing affordable and enticing activities may reduce SB through providing opportunities for social interaction, peer support, and increased community involvement. Provider-level interventions could target healthcare professionals who interact with older adults such as physiotherapists. Older adults are unaware of their SB, the health consequences of SB, and the benefits of reducing their SB [63, 70, 76, 82]. With older adults valuing receiving information from credible sources [66, 67], provider-level interventions could target screening of SB to identify older adults with excessive SB and provide education.

### Intervention duration

Interventions to reduce SB were evenly distributed with regards to intervention duration and ranged from 2 to 36 weeks. Long-term interventions were more effective than short-term and medium-term interventions, however, there was little indication of whether intervention effects were maintained as only three SB interventions incorporated medium or long-term follow-ups following intervention cessation [45, 53, 85]. From the qualitative review, older adults expressed the deeply ingrained nature of SB, and that meaningful change may not be maintained [58, 63, 67–69, 77, 84, 88]. Including follow-ups following cessation can provide information on whether interventions effects are maintained, and the act of following up can promote further adherence [66, 67, 89].

### Intervention content

This review identified that interventions to reduce SB were slightly more effective at reducing SB than interventions also aimed to increase PA when device-measured (−11.61 min/day vs. −10.78 min/day) and self-reported (−83.65 min/day vs. 9.75 min/day), echoing the findings of previous reviews in adults [110, 111]. This may be attributable to increased compensatory sedentary time in SB due to completing more MVPA, or varying levels of emphasis placed on reducing sedentary behaviour in interventions that aimed to increase PA and reduce SB.

Additionally, the needs of older adults were also underrepresented in intervention content. Social support was frequently requested by older adults (Table 4) yet was incorporated in less than half of the included interventions. Similarly, receiving information from credible sources, such as healthcare professionals, was valued, but only seven interventions included this BCT. Using ≥ 10 BCTs appeared more effective than using 1–10 BCTs, supporting the findings of a review by Curran et al. [112], which explored interventions to reduce SB in healthy adults, and the theory of additive effects through combining BCTs [41]. More precise descriptions of interventions are needed, as only three of the included interventions to reduce SB [43, 44, 85], provided their incorporated BCTs. Future research should incorporate intervention components valued by older adults and clearly document the intervention components employed.

Evidence from Crombie et al. [45] and Maher et al. [50] suggests that domain-specific reductions of SB are possible, with considerable reductions in television viewing time (self-reported) achieved while maintaining levels of mentally active activities such as computer use. In the wider literature, engaging in mentally active sedentary behaviours such as socialising, computer-use or reading is associated with fewer difficulties with ADLs and higher cognitive functioning than older adults who engaged in mentally passive activities such as watching television [107]. Targeting reductions in mentally passive SB may therefore be both a more acceptable and a more effective strategy for older adults.

### Strengths

This was the first mixed-method review conducted on interventions to reduce SB in older adults, and through the integration of the qualitative and quantitative findings, a novel interpretation of the appropriateness of existing interventions is provided. Secondly, the charting intervention of BCTs incorporated can further our understanding of the approaches taken and provide a preliminary exploration of the efficacy of these interventions. Finally, this review employed a rigorous methodology, a large search strategy that yielded over 50,000 articles for screening and included articles that had a low risk of bias.

### Limitations

Only studies published in English were eligible for inclusion. The resulting sample consisted mainly of white females aged 65–74, limiting the generalisability of review findings across the wider older adult population, particularly the oldest old. The qualitative synthesis is a secondary interpretation of quotes provided in the primary studies which may introduce bias. The decision to include studies conducted in specific populations, such as cancer survivors, may have introduced heterogeneity. However, this inclusion may provide a more representative view as two-thirds of community-dwelling older adults live with chronic conditions [113]. Lastly, active controls may have inflated the pooled control results, limiting the effect size. Active controls were also incorporated in the review by Chastin et al. [11], who reasoned that physical activity interventions have been shown to not change SB in older adults [110]. However, the results from our review suggest that interventions to increase PA and reduce SB resulted in comparable reductions in sedentary time (−11.27 min/day vs. −10.78 min/day).

## Conclusion

Interventions to reduce SB in community-dwelling older adults can achieve their intended purpose but may not reach the MCID to achieve important effects on key outcomes when device-measured SB data is used. Existing trials are characterised by limited information on medium-long term outcomes, and outcomes important to older people, such as HRQoL or pain, which have not been routinely measured. Further research should adopt inclusive recruitment strategies to target underrepresented groups such as adults aged ≥ 75 years, incorporate older adults’ views in intervention design and outcome measurement selection, and explore provider-level and system-level interventions.

## Supplementary Information

Supplementary Material 1.

Supplementary Material 2.

## Data Availability

Available from the corresponding author upon reasonable request

## Abbreviations

BCT: Behaviour Change Technique
CI: Confidence interval
COM-B: Capability, Opportunity Motivation - behaviour
HRQoL: Health-related quality of life
MCID: Minimal clinically important difference
METS: Metabolic equivalent of taskMMAT: Mixed Method Appraisal Tool
MVPA: Moderate-vigorous physical activity
PA: Physical activity
RCT: Randomised controlled trial
SB: Sedentary behaviour
SD: Standard deviation
