# Supplementary material for: Interventions to reduce sedentary behaviour in community-dwelling older adults: a mixed-method review
Source: Int J Behav Nutr Phys Act. 2025 Nov 12;22:141. doi: 10.1186/s12966-025-01835-3 (PMC12606893; doi:10.1186/s12966-025-01835-3)
Supplement: Supplementary file 1 — Supplementary Material 1. [file 12966_2025_1835_MOESM1_ESM.docx]

**Medline (Ovid) Qualitative Search**

1 Sedentary behavior/

2 (sedentary or sitting or sedentariness or sedentarism).ti.

3 ((sedentary or sitting or seated) adj5 (behavio* or lifestyle or life-style or pattern* or leisure or time or bout* or prolonged)).tw,kf.

4 (sedentary adj3 (adult? or men or women or males or females or individual? or people or person or population?)).tw,kf.

5 physical* inactiv*.tw,kf.

6 physical activity.tw,kf.

7 ((leisure time or life style or lifestyle) adj5 (physical* activ* or passive or inactiv* or physical fitness or walking or walk)).tw,kf.

8 ("sit less" or "sitting less").tw,kf.

9 screen time/

10 sitting position/

11 (time adj5 (computer* or television or tv or screen)).ti.

12 ((watch* or view*) adj5 (television or tv)).tw,kf.

13 or/1-12 [sedentary behaviour]

14 (older adj (adult* or people or person? or population* or m#n or wom#n or male* or female* or patient* or individual?)).tw,kf.

15 (geriatr* or elderly or seniors or senior).tw,kf.

16 (retired or retirement).tw,kf.

17 exp Aged/

18 Geriatrics/

19 (age? adj3 (over or older) adj2 (5# or 6# or 7# or 8# or 9#)).tw.

20 sexagenarian.tw,kf.

21 septuagenarian.tw,kf.

22 octogenarian.tw,kf.

23 nonagenerian.tw,kf.

24 centenarian.tw,kw.

25 gerontolog*.tw,kf.

26 (">=5# years old" or ">5#years old").tw.

27 (">=6# years old" or ">6#years old").tw.

28 (">=7# years old" or ">7#years old").tw.

29 (">=8# years old" or ">8#years old").tw.

30 (">=9# years old" or ">9#years old").tw.

31 or/14-30 [older people]

32 interview*.tw,kf.

33 qualitative.tw,kf.

34 exp qualitative research/

35 theme*.tw,kf.

36 (survey* or questionnaire* or "focus group*").tw,kf.

37 "Surveys and Questionnaires"/

38 Focus Groups/

39 or/32-38 [qualitative research filter]

40 13 and 31 and 39 [sedentary behaviour and elderly and qualitative research filter]

41 (exp Child/ or Adolescent/ or exp Infant/) not exp Adult/

42 40 not 41 [adult studies only]

**Medline (Ovid) quantitative Search**

1 Sedentary behavior/

2 (sedentary or sitting or sedentariness or sedentarism).ti.

3 ((sedentary or sitting or seated) adj5 (behavio* or lifestyle or life-style or pattern* or leisure or time or bout* or prolonged)).tw,kf.

4 (sedentary adj3 (adult? or men or women or males or females or individual? or people or person or population?)).tw,kf.

5 physical* inactiv*.tw,kf.

6 physical activity.tw,kf.

7 ((leisure time or life style or lifestyle) adj5 (physical* activ* or passive or inactiv* or physical fitness or walking or walk)).tw,kf.

8 ("sit less" or "sitting less").tw,kf.

9 screen time/

10 sitting position/

11 (time adj5 (computer* or television or tv or screen)).ti.

12 ((watch* or view*) adj5 (television or tv)).tw,kf.

13 or/1-12 [sedentary behaviour]

14 (older adj (adult* or people or person? or population* or m#n or wom#n or male* or female* or patient* or individual?)).tw,kf.

15 (geriatr* or elderly or seniors or senior).tw,kf.

16 (retired or retirement).tw,kf.

17 exp Aged/

18 Geriatrics/

19 (age? adj3 (over or older) adj2 (5# or 6# or 7# or 8# or 9#)).tw.

20 sexagenarian.tw,kf.

21 septuagenarian.tw,kf.

22 octogenarian.tw,kf.

23 nonagenerian.tw,kf.

24 centenarian.tw,kw.

25 gerontolog*.tw,kf.

26 (">=5# years old" or ">5#years old").tw.

27 (">=6# years old" or ">6#years old").tw.

28 (">=7# years old" or ">7#years old").tw.

29 (">=8# years old" or ">8#years old").tw.

30 (">=9# years old" or ">9#years old").tw.

31 or/14-30 [older people]

32 randomized controlled trial.pt.

33 controlled clinical trial.pt.

34 randomized.ab.

35 placebo.ab.

36 drug therapy.fs.

37 randomly.ab.

38 trial.ab.

39 groups.ab.

40 32 or 33 or 34 or 35 or 36 or 37 or 38 or 39

41 exp animals/ not humans.sh.

42 40 not 41

43 40 not 41 [Cochrane sensitivity maximising RCT filter]

44 13 and 31 and 43 [sedentary behaviour and elderly and RCTs]

45 (exp Child/ or Adolescent/ or exp Infant/) not exp Adult/

46 44 not 45 [adult only studies]
