# Supplementary material for: Interventions to reduce sedentary behaviour in community-dwelling older adults: a mixed-method review
Source: Int J Behav Nutr Phys Act. 2025 Nov 12;22:141. doi: 10.1186/s12966-025-01835-3 (PMC12606893; doi:10.1186/s12966-025-01835-3)
Supplement: Supplementary file 2 — Supplementary Material 2. [file 12966_2025_1835_MOESM2_ESM.docx]

**Secondary Outcomes**

The effects of interventions which aimed to reduce SB and interventions which aimed to increase PA and SB are summarised below.

1. **Interventions to Reduce SB vs Interventions to Increase PA and Reduce SB**

**
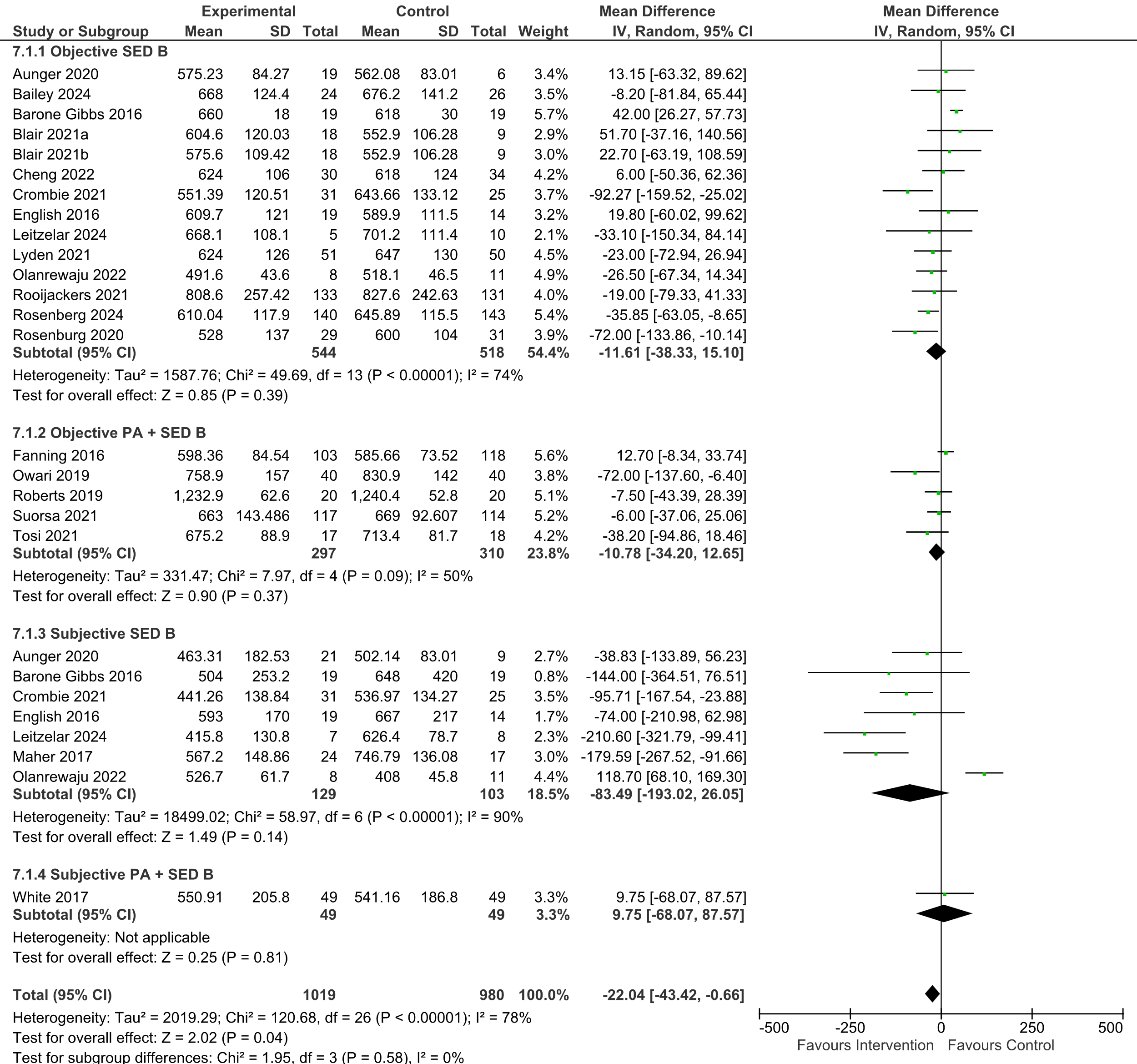
**

1. **Length of Interventions**

**
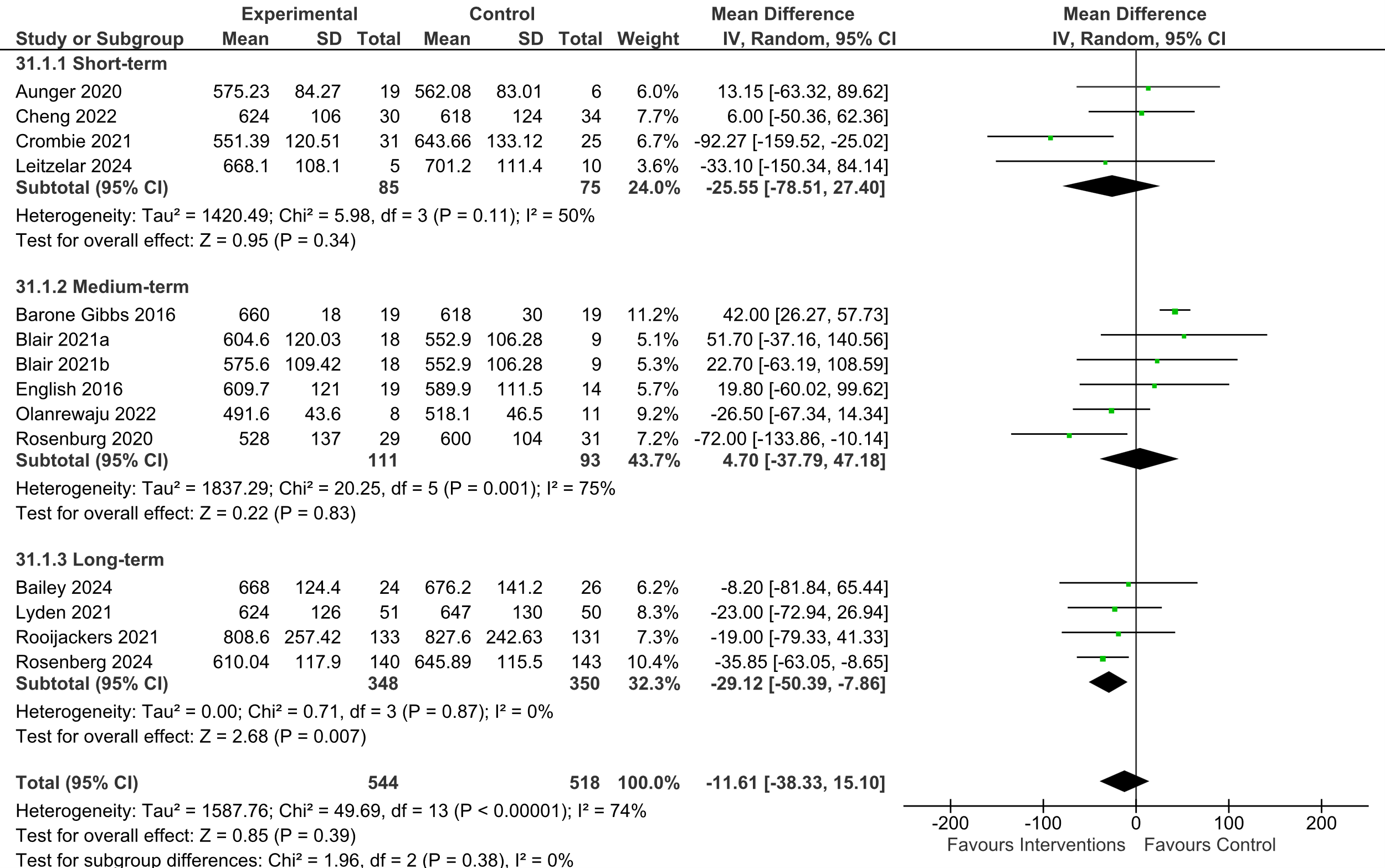
**

1. **Number of BCTs**

**
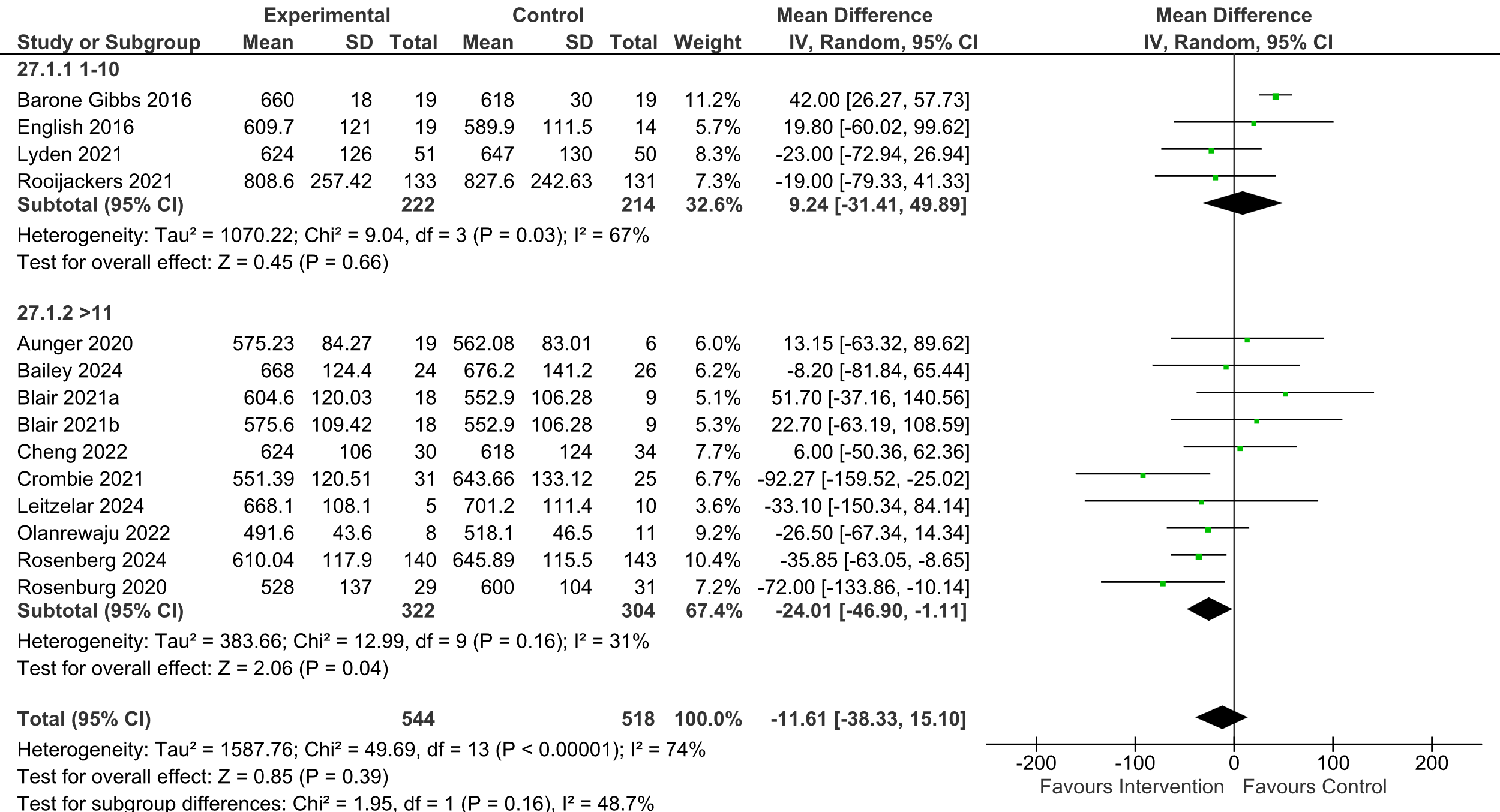
**

1. **Sit-to-Stand Transitions**

**
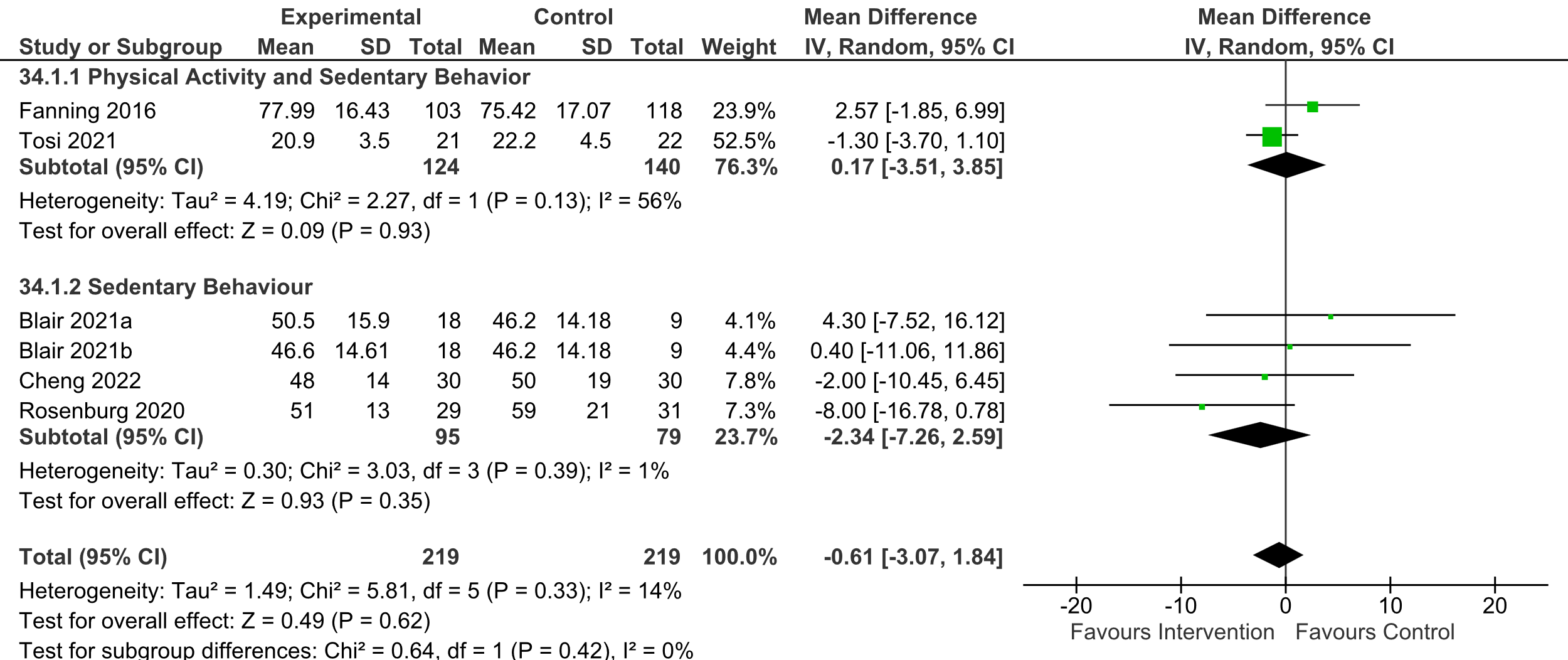
**The results of five studies which explored the impact of interventions on sit-to-stand (STS) transitions were pooled (1-5). Interventions were less effective than controls at increasing sit-to-stand transitions (-0.61 transitions 95% CI -3.07 to 1.84, P=0.62, I^2^**=**14%). Interventions to reduce SB observed a reduction in STS transitions (-2.34 transitions 95%CI -7.26 to 2.59, P=0.35, I^2^**=**1%), whereas interventions to increase PA and reduce SB observed a slight increase (0.17 95% CI -3.51 to 3.85, P=0.13, I^2^=56%).

(-11.61 mins/day 95% CI -38.33 to 15.10, P=0.39 -27.53 mins/day (95% CI -57.43 to 2.37, P =0.07, I² =82%)

1. **Sedentary Breaks**

The results of five studies which explored the impact of interventions on sedentary breaks were pooled (6-10). Interventions were more effective than controls at increasing the number of sedentary breaks (1.57 breaks 95% CI -2.44 to 5.57, P=0.44, I^2^=37%). **
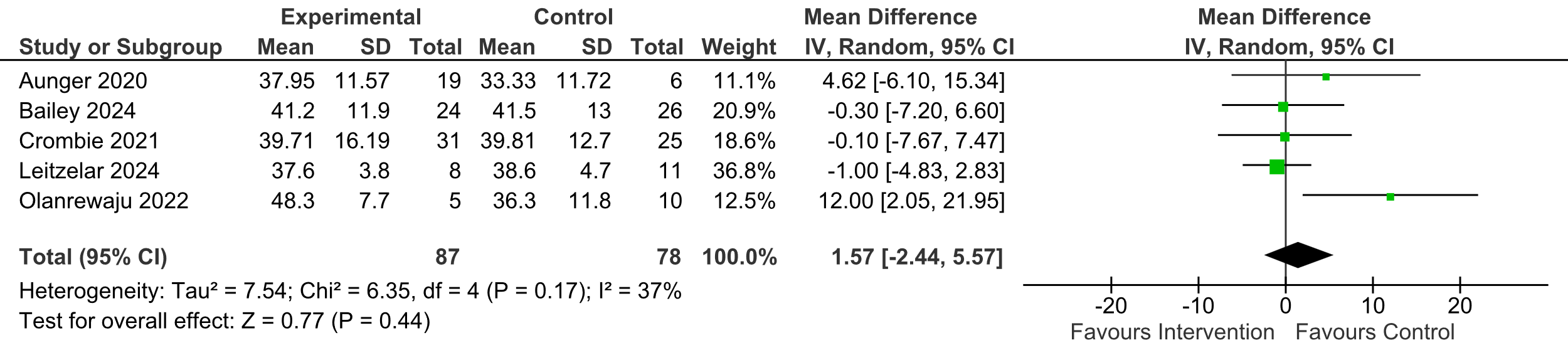
**

1. **Prolonged Sedentary Bouts (>30 Minutes)**

The results of six studies which explored the impact of interventions on reducing the duration of prolonged sedentary bouts (>30 minutes) were pooled (1, 2, 6, 7, 10, 11). Interventions were more effective than controls at reducign the duration of prolonged sedentary bouts (-6.15 mins 95% CI-29.78 to 17.48, P=0.61, I^2^=0%).

**
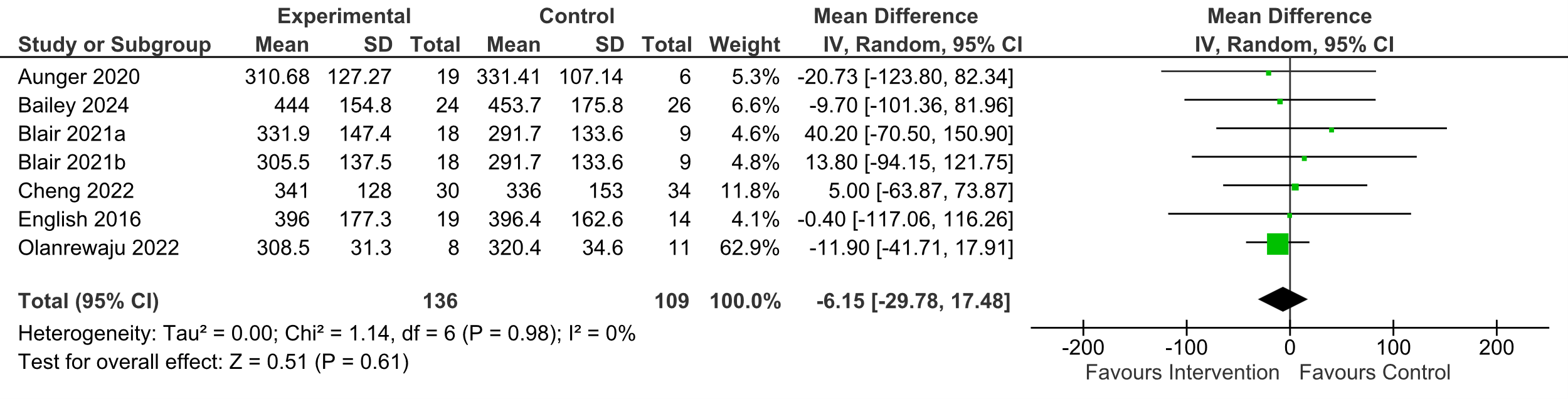
**

1. **Moderate-Vigorous Physical Activity**

The results of eight studies which measured the effects of interventions on increasing MVPA were pooled (1, 2, 8, 9, 11-14). Interventions were less effective than controls at increasing MVPA (-1.04 mins/day 95% CI-11.48 to 9.40, P=0.85), with both SB interventions (1, 2, 8, 9, 11, 12) and PA and SB interventions (13, 14) observing reductions in MVPA (-0.70 mins/day 95% CI-10.45 to 9.04 P=0.89 vs -5.62mins/day -69.18 to 57.93, P=0.86).


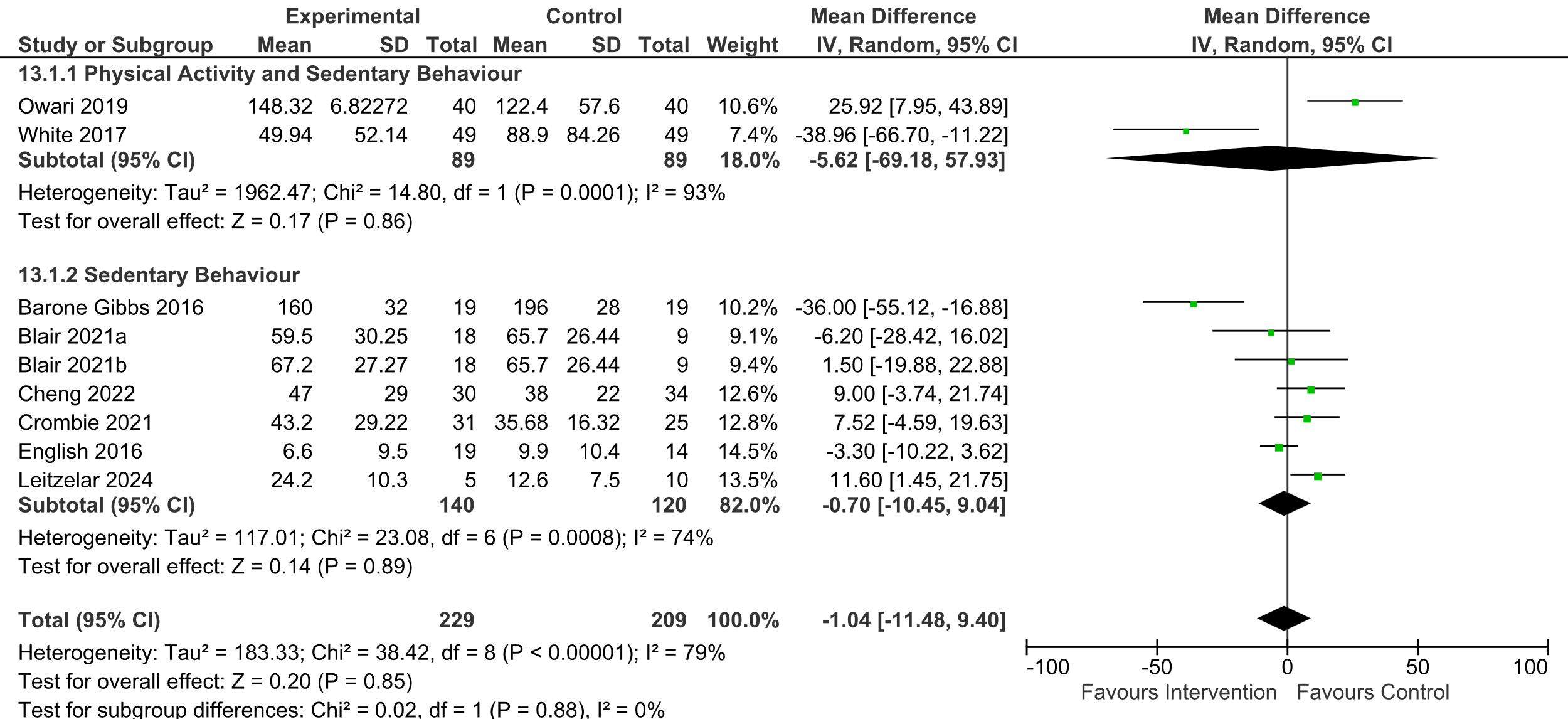


1. **Light-Intensity Physical Activity**

The results of five studies which explored the impact of interventions to reduce SB on LIPA were pooled (1, 2, 8, 9, 12). A reduction of 10.97 mins/day (95% CI -25.76 to 3.81, P=0.15) was observed when compared to the controls.


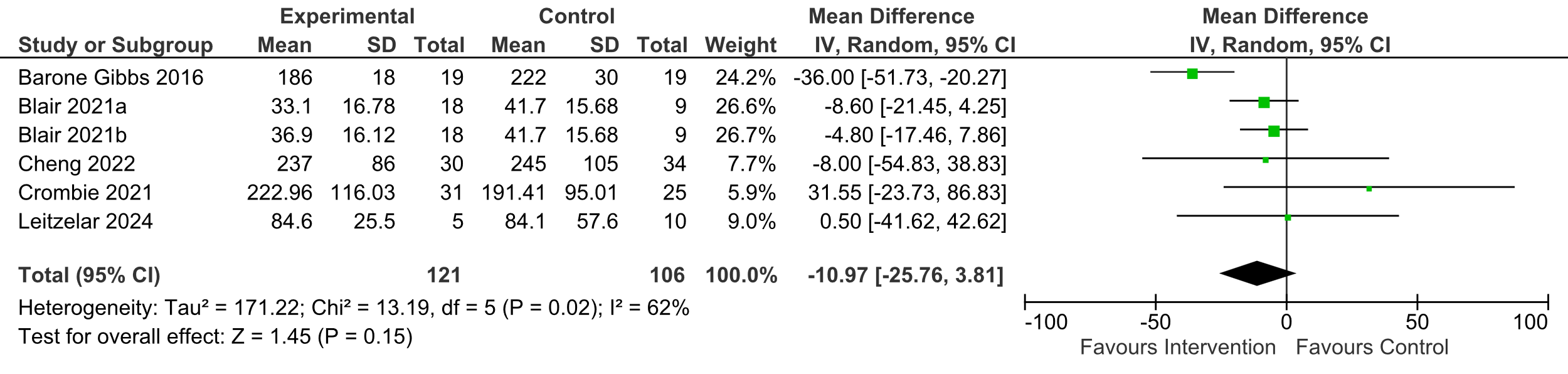


1. **Step Counts**

The results of 11 studies which explored the impact of interventions on step counts were pooled (1-3, 6, 7, 10, 13, 15-17). Interventions were more effective than controls at improving step counts (550 steps, 95% CI 160 to 950, P=0.006). Interventions which aimed to improve PA and reduce SB were more effective than interventions which aimed to reduce SB (840 steps, 95% CI 20 to 1660 P=0.05 vs 460 steps 95% CI 10 to 920, P=0.04).


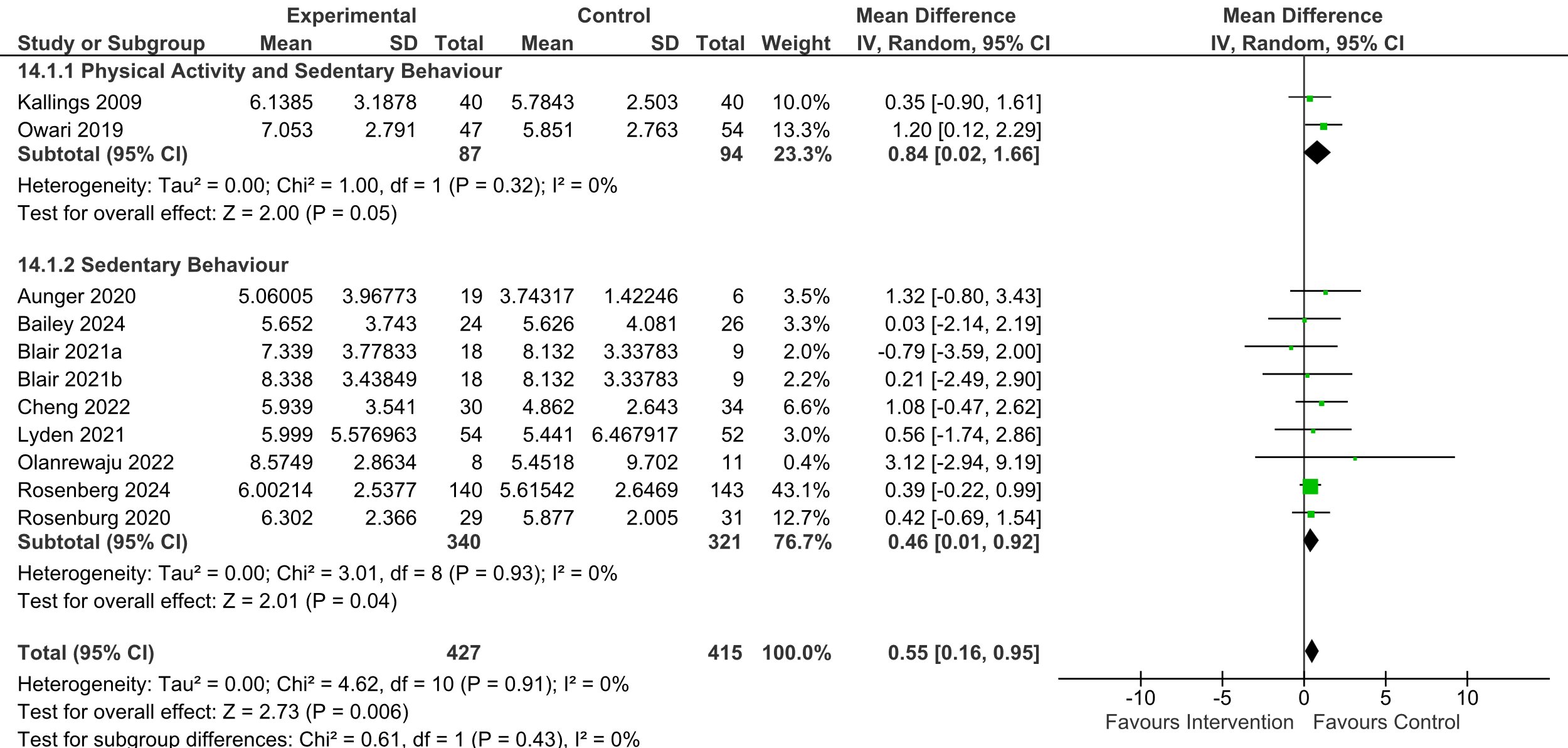


1. **6 Minute Walk Test**

Three studies used the 6 Minute Walk Test (2, 15, 18) to explore the impact of interventions on walking distance. Studies by Cheng et al.(2) and Lyden et al.(15) observed comparable increases in walking distance when compared to the controls ( 10 steps 95% CI -8 to 28 vs MD 18 steps 95% CI 5 to 32) and (MD 26 Steps 95% CI 14 to 39 vs MD 25 steps 95% CI 12 to 37). Conversely, Roberts et al.(18) observed a reduction of 15m (95% CI −40.44 to 10.29) when compared to controls.

1. **Short Physical Performance Battery**

The results of eight studies which explored the impact of interventions on the Short Physical Performance Battery total scores were pooled (1, 3, 6, 7, 9, 12, 19). Interventions were marginally more effective than controls at improving total SPPB scores (0.08 95% CI -0.23 to 0.40, P=0.84). Interventions to reduce SB observed an increase of 0.04 (95% CI -0.34 to 0.42, P=0.84) (1, 3, 6, 7, 9, 12, 18, 19), whereas Roberts et al.(18) observed an increase of 0.20 (95% CI-0.67 to 1.07, P=0.65).

**
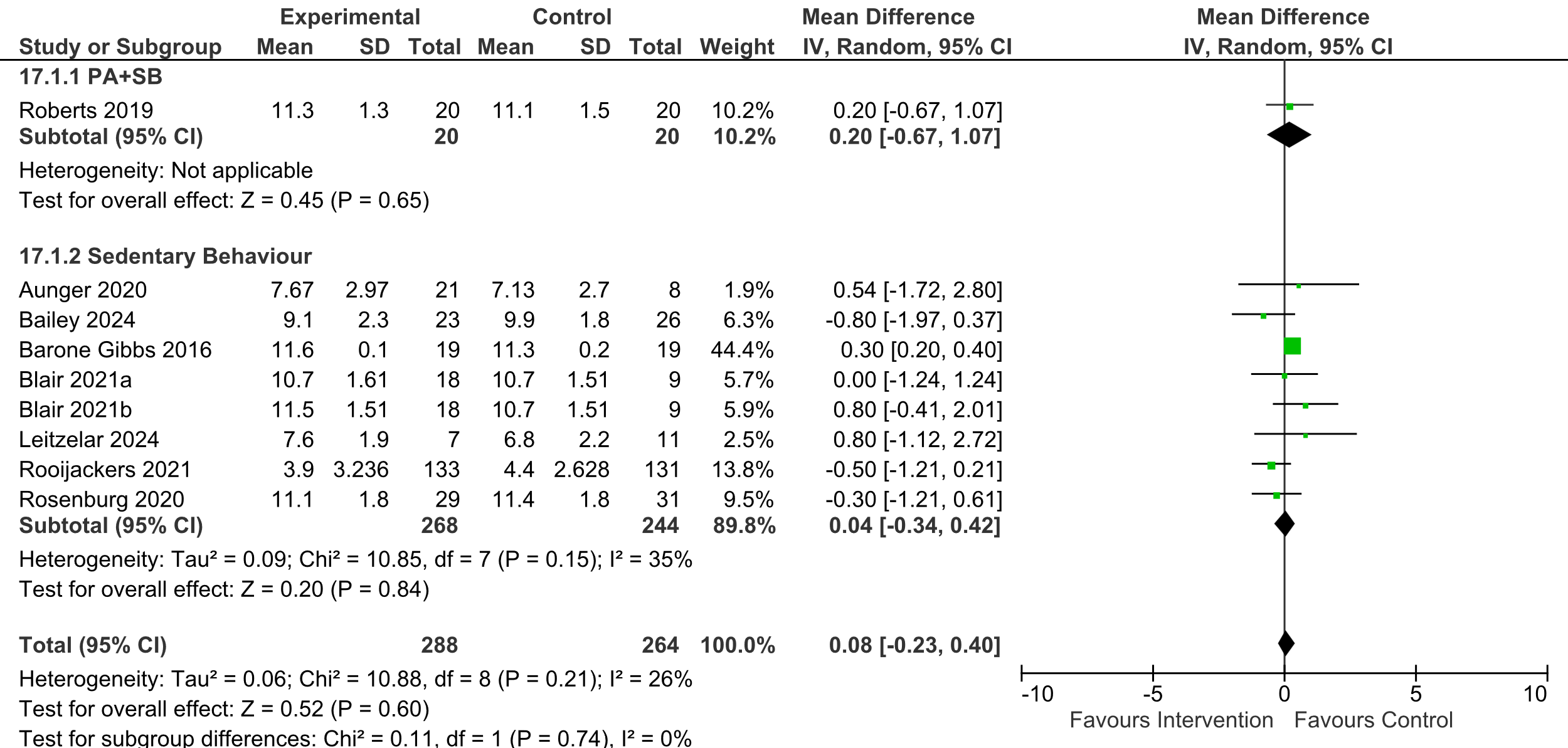
**

1. **Body Mass Index**

The results of seven studies which explored the impacts of interventions to reduce SB on BMI were pooled (3, 6, 7, 13, 15-17). Interventions were more effective than controls at reducing BMI (-0.67 95% CI -1.25 to -0.09, P=0.02). Interventions to reduce SB observed a reduction of -0.19 (95% CI-0.96 to 0.59. P=0.64) (3, 6, 7, 15, 16) when pooled.


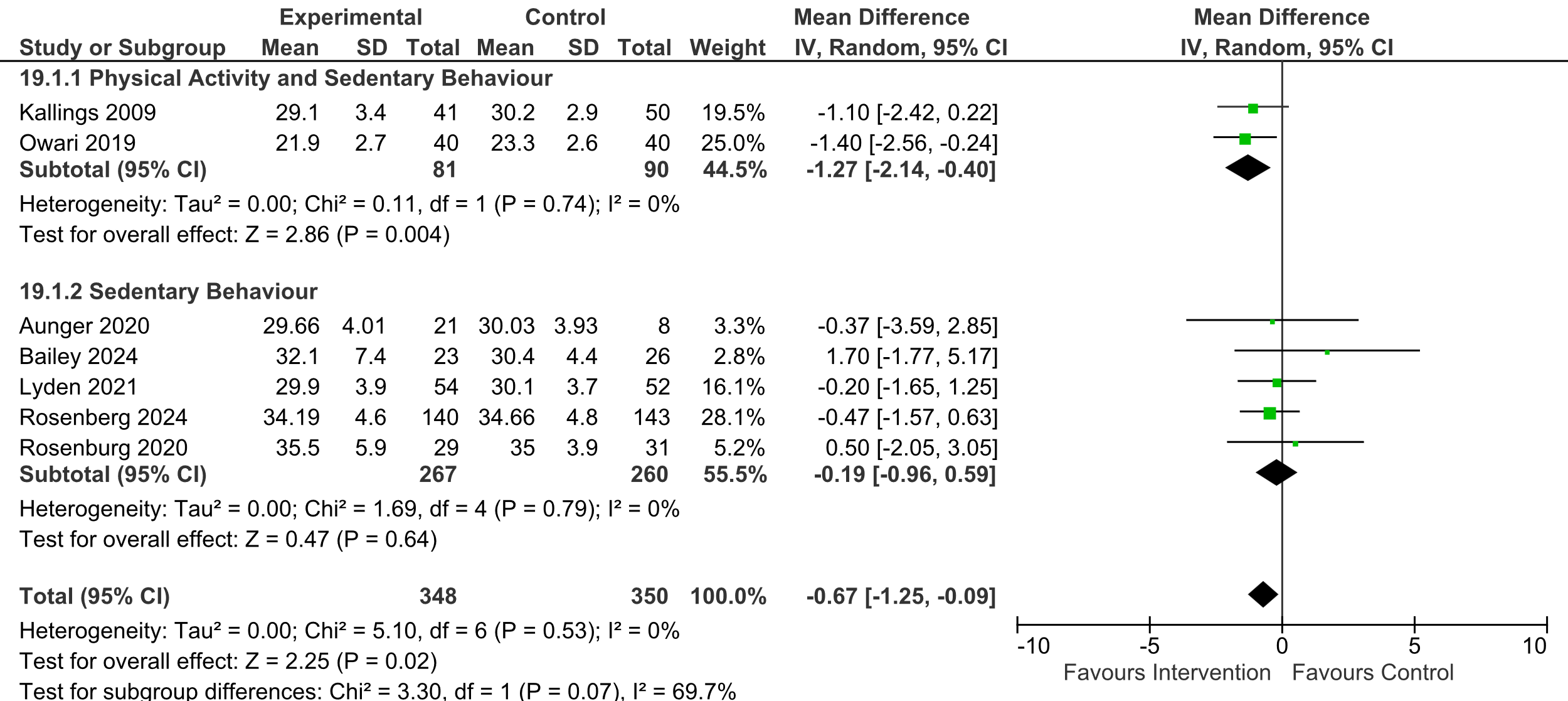


1. **Waist Circumference**

The results of five studies which explored the impact of interventions on reducing waist circumference were pooled (3, 15-18). Interventions were more effective than control at reducing waist circumference (-0.61cm 95% CI -2.25 to 1.03, P=0.46). Interventions which aimed to reduce SB (3, 15, 16) observed greater reductions than interventions which also aimed to increase physical activity (17, 18) when compared (-1.04cm 95% CI -3.37 to 1.29, P= 0.38 vs -0.27cm 95% -3.59, 3.05, P=0.87).


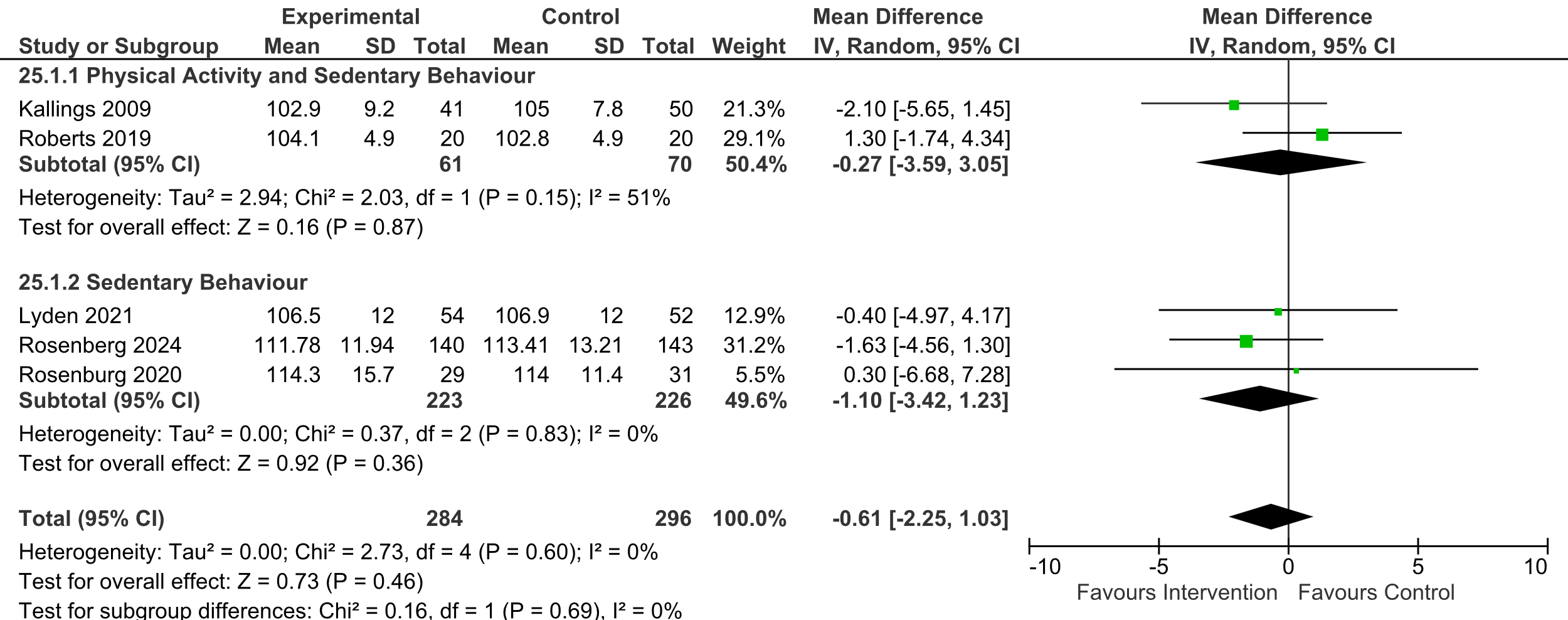


1. **Blood Pressure**

The results of five studies which explored the impact of interventions on blood pressure were pooled (3, 12, 16-18). Interventions were more effective than controls at reducing systolic blood pressure (-1.82 mmHg 95% CI -4.33 to 0.68, P=0.15) and diastolic blood pressure (-0.14 mmHg 95% CI-2.84 to 2.56, P=0.92). Interventions to increase PA and reduce SB observed a slightly greater reduction in systolic blood pressure (-3.97 mmHg 95% CI -13.06 to 5.12, P=0.39 vs -2.57 mmHg 95% CI-7.45 to 2.30, P=0.30). Similarly, interventions to increase PA and reduce SB observed a greater reduction in diastolic blood pressure than interventions to reduce SB (-1.25 mmHg, 95% CI -1.98 to -0.52, P=0.0008 vs 0.60 mmHg 95% CI -4.43 to 5.64, P=0.81).

**
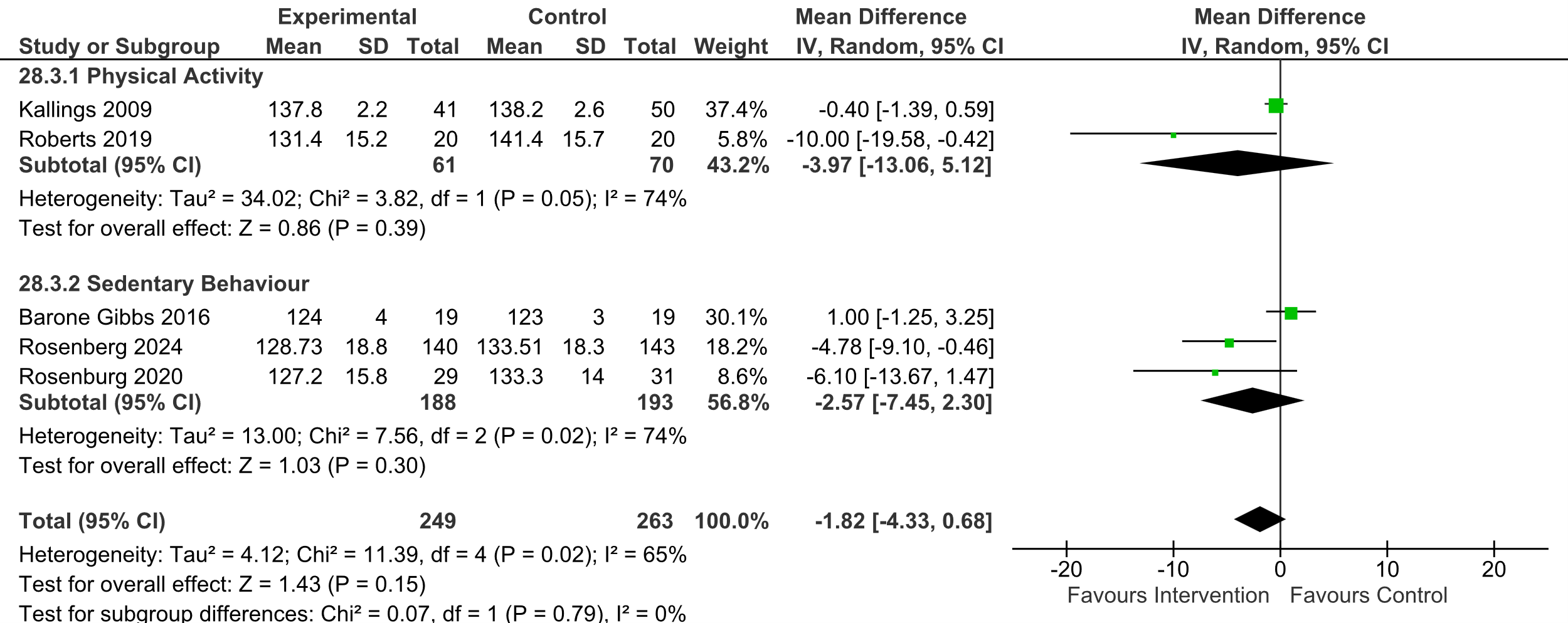
**

**
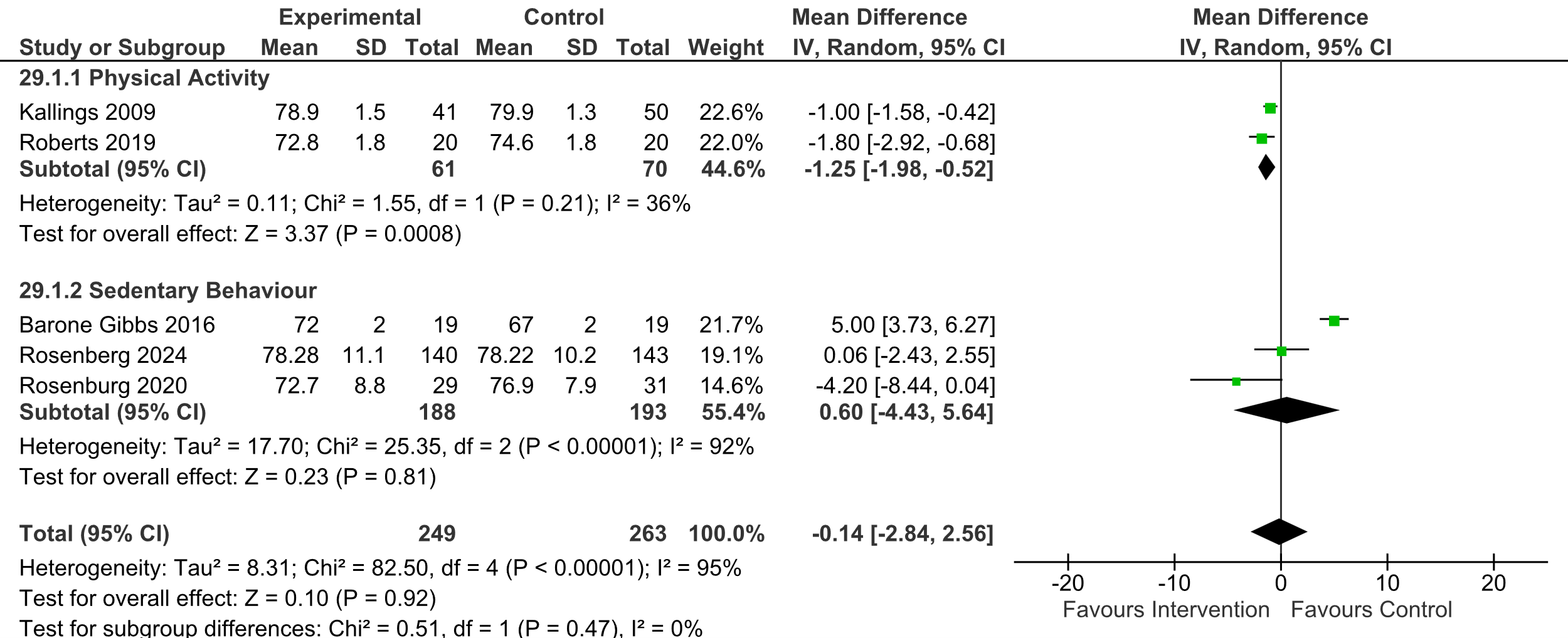
**

1. **Health-Related Quality of Life**

Six studies assessed health-related quality of life (HRQoL) using validated measures (1, 6, 8-10, 12), including four using the SF-36 (1, 8, 9, 12) and two using the EQ-5D Visual Analogue Scale (VAS) (6, 10). Overall, findings were mixed, with some evidence of domain-specific improvements but limited statistically significant between-group effects.

Among the four studies using the SF-36, three reported clinically meaningful or statistically significant improvements in select subdomains. Crombie et al.(8) found significant group × time interactions favouring the intervention group for vitality, general health, and role limitations due to physical and emotional health, with small to moderate effect sizes (d = 0.29–0.56) sustained at follow-up. Barone Gibbs et al.(12) observed a significant improvement in bodily pain in the Sit Less group compared to the Get Active group, while Leitzelar et al.(9) reported a moderate improvement in emotional role functioning in the intervention group (g = 0.54). Blair et al.(1) reported no statistically significant between-group differences, although several subscales exceeded the minimally clinically important difference threshold (≥4 points), suggesting potential improvements in general health, role physical, social functioning, and vitality in the health coaching arm.

The two studies using the EQ-5D VAS showed minimal and non-significant changes in self-rated health. Aunger et al.(6) reported a small, non-significant increase in VAS scores in the intervention group (+5.29 points) and a slight decrease in the control group (–1.11 points). Olanrewaju et al.(10) found negligible changes in VAS scores in both groups (–2.8 and +1.1 points, respectively), with no statistically significant differences.

Collectively, these findings suggest that interventions aiming to reduce sedentary behaviour may lead to modest, domain-specific improvements in quality of life, particularly in mental health and vitality-related domains, though results are variable and often not statistically robust.

1. **Cardiometabolic Biomarkers**

Four studies assessed the impact of interventions on cardiometabolic biomarkers, including total cholesterol, HDL, LDL, triglycerides, glucose, and glycated haemoglobin (HbA1c) (3, 6, 17, 18). Overall, findings were mixed, with three studies reporting no significant between-group differences and one study reporting modest, statistically significant improvements favouring the intervention.

Aunger et al.(6) and Rosenberg et al.(3) both reported no statistically significant changes in any biomarker between groups. Small between-group differences in total cholesterol (–0.09 to –0.25 mmol/L), LDL (–0.03 to +0.08 mmol/L), HDL (–0.01 to –0.09 mmol/L), and triglycerides (–0.11 to +0.03 mmol/L) were observed, but none were clinically meaningful or statistically significant. Glucose and HbA1c changes were also minimal.

Roberts et al.(18) reported no significant differences in adjusted mean changes for any lipid or glucose-related outcome. Between-group differences in total cholesterol (+0.13 mmol/L), LDL (+0.12 mmol/L), HDL (–0.05 mmol/L), and HbA1c (+0.11%) were small and not statistically significant.

In contrast, Kallings et al.(17) found significant between-group improvements in HbA1c (–0.25%; 95% CI: –0.37 to –0.12) and total cholesterol (–0.3 mmol/L; 95% CI: –0.7 to –0.01) favouring the intervention group at 6 months. Within-group reductions were also observed in triglycerides (–13.1%) and glucose (–3.4%) in the intervention group, although between-group differences for these markers did not reach statistical significance.

Together, these findings suggest that while most interventions had little impact on cardiometabolic biomarkers, there is some evidence that longer-duration or more intensive interventions may produce modest improvements in specific outcomes such as cholesterol and HbA1c.

**References**

1. Blair CK, Harding E, Wiggins C, Kang H, Schwartz M, Tarnower A, et al. A Home-Based Mobile Health Intervention to Replace Sedentary Time With Light Physical Activity in Older Cancer Survivors: Randomized Controlled Pilot Trial. The Journal of Medical Internet Research Cancer. 2021;7(2):e18819.

2. Cheng SWM, Alison J, Stamatakis E, Dennis S, McNamara R, Spencer L, et al. Six-week behaviour change intervention to reduce sedentary behaviour in people with chronic obstructive pulmonary disease: a randomised controlled trial. Thorax. 2022;77(3):231-8.

3. Rosenberg DE, Anderson ML, Renz A, Matson TE, Lee AK, Greenwood-Hickman MA, et al. Reducing Sitting Time in Obese Older Adults: The I-STAND Randomized Controlled Trial. Journal of Aging and Physical Activity. 2020:1-11.

4. Fanning J, Porter G, Awick EA, Wójcicki TR, Gothe NP, Roberts SA, et al. Effects of a DVD-delivered exercise program on patterns of sedentary behavior in older adults: a randomized controlled trial. Preventive Medicine Reports. 2016;3:238-43.

5. Tosi FC, Lin SMM, Gomes GC, Aprahamian I, Nakagawa NK, Viveiro L, et al. A multidimensional program including standing exercises, health education, and telephone support to reduce sedentary behavior in frail older adults: Randomized clinical trial. Experimental Gerontology. 2021;153.

6. Aunger JA, Greaves CJ, Davis ET, Asamane EA, Whittaker AC, Greig CA. A novel behavioural INTErvention to REduce Sitting Time in older adults undergoing orthopaedic surgery (INTEREST): results of a randomised-controlled feasibility study. Aging Clinical and Experimental Research. 2020;32(12):2565-85.

7. Bailey DP, Harper JH, Kilbride C, McGowan LJ, Victor C, Brierley ML, et al. The frail-LESS (LEss sitting and sarcopenia in frail older adults) remote intervention to improve sarcopenia and maintain independent living via reductions in sedentary behaviour: findings from a randomised controlled feasibility trial. BMC Geriatrics. 2024;24(1):747.

8. Crombie KM, Leitzelar BN, Almassi NE, Mahoney JE, Koltyn KF. The Feasibility and Effectiveness of a Community-Based Intervention to Reduce Sedentary Behavior in Older Adults. Journal of Applied Gerontology. 2021:073346482098791.

9. Leitzelar BN, Almassi NE, Andreae SJ, Winkle-Wagner R, Cadmus-Bertram L, Columna L, et al. Intervening to reduce sedentary behavior among African American elders: the "Stand Up and Move More" intervention. Health Promotion Perspectives. 2024;14(2):148-60.

10. Olanrewaju O, Carmichael C, Wallis J, Smith L. Reducing sedentary behaviour and cognitive function in older people with Mild Cognitive Impairment: Results of a randomized feasibility study. Aging and Health Research. 2022;2(1):100057.

11. English C, Healy GN, Olds T, Parfitt G, Borkoles E, Coates A, et al. Reducing Sitting Time After Stroke: A Phase II Safety and Feasibility Randomized Controlled Trial. Archives of Physical Medicine and Rehabilitation. 2016;97(2):273-80.

12. Barone Gibbs B, Brach JS, Byard T, Creasy S, Davis KK, McCoy S, et al. Reducing Sedentary Behavior Versus Increasing Moderate-to-Vigorous Intensity Physical Activity in Older Adults: A 12-Week Randomized, Clinical Trial. Journal of Aging and Health. 2016.

13. Owari Y, Suzuki H, Miyatake N. "Active Guide" Brochure Reduces Sedentary Behavior of Elderly People: A Randomized Controlled Trial. Acta Medica Okayama. 2019;73(5):427-32.

14. White I, Smith L, Aggio D, Shankar S, Begum S, Matei R, et al. On Your Feet to Earn Your Seat: pilot RCT of a theory-based sedentary behaviour reduction intervention for older adults. Pilot and Feasibility Studies. 2017;3(1).

15. Lyden K, Boucher R, Wei G, Zhou N, Christensen J, Chertow GM, et al. Targeting Sedentary Behavior in CKD: A Pilot and Feasibility Randomized Controlled Trial. Clinical Journal of the American Society of Nephrology. 2021.

16. Rosenberg DE, Zhu W, Greenwood-Hickman MA, Cook AJ, Florez Acevedo S, McClure JB, et al. Sitting Time Reduction and Blood Pressure in Older Adults: A Randomized Clinical Trial. JAMA Network Open. 2024;7(3):e243234.

17. Kallings LV, Johnson JS, Fisher RM, Faire UD, Ståhle A, Hemmingsson E, et al. Beneficial effects of individualized physical activity on prescription on body composition and cardiometabolic risk factors: results from a randomized controlled trial. European Journal of Cardiovascular Prevention & Rehabilitation. 2009;16(1):80-4.

18. Roberts LM, Jaeger BC, Baptista LC, Harper SA, Gardner AK, Jackson EA, et al. Wearable Technology To Reduce Sedentary Behavior And CVD Risk In Older Adults: A Pilot Randomized Clinical Trial. Clinical Interventions in Aging. 2019;Volume 14:1817-28.

19. Rooijackers TH, Kempen GIJM, Zijlstra GAR, van Rossum E, Koster A, Lima Passos V, et al. Effectiveness of a reablement training program for homecare staff on older adults' sedentary behavior: A cluster randomized controlled trial. Journal of the American Geriatrics Society. 2021;69(9):2566-78.
